# Stratification by Tumor Grade Groups in a Holistic Evaluation of Machine Learning for Brain Tumor Segmentation

**DOI:** 10.3389/fnins.2021.740353

**Published:** 2021-10-06

**Authors:** Snehal Prabhudesai, Nicholas Chandler Wang, Vinayak Ahluwalia, Xun Huan, Jayapalli Rajiv Bapuraj, Nikola Banovic, Arvind Rao

**Affiliations:** ^1^Computer Science and Engineering, University of Michigan, Ann Arbor, MI, United States; ^2^Computational Medicine and Bioinformatics, Michigan Medicine, Ann Arbor, MI, United States; ^3^Perelman School of Medicine at the University of Pennsylvania, Philadelphia, PA, United States; ^4^Mechanical Engineering, University of Michigan, Ann Arbor, MI, United States; ^5^Department of Radiology, University of Michigan, Ann Arbor, MI, United States; ^6^Department of Biostatistics, University of Michigan, Ann Arbor, MI, United States; ^7^Department of Radiation Oncology, University of Michigan, Ann Arbor, MI, United States; ^8^Department of Biomedical Engineering, University of Michigan, Ann Arbor, MI, United States

**Keywords:** medical AI, evaluation, brain imaging, segmentation, GBM, LGG

## Abstract

Accurate and consistent segmentation plays an important role in the diagnosis, treatment planning, and monitoring of both High Grade Glioma (HGG), including Glioblastoma Multiforme (GBM), and Low Grade Glioma (LGG). Accuracy of segmentation can be affected by the imaging presentation of glioma, which greatly varies between the two tumor grade groups. In recent years, researchers have used Machine Learning (ML) to segment tumor rapidly and consistently, as compared to manual segmentation. However, existing ML validation relies heavily on computing summary statistics and rarely tests the generalizability of an algorithm on clinically heterogeneous data. In this work, our goal is to investigate how to holistically evaluate the performance of ML algorithms on a brain tumor segmentation task. We address the need for rigorous evaluation of ML algorithms and present four axes of model evaluation—diagnostic performance, model confidence, robustness, and data quality. We perform a comprehensive evaluation of a glioma segmentation ML algorithm by stratifying data by specific tumor grade groups (GBM and LGG) and evaluate these algorithms on each of the four axes. The main takeaways of our work are—(1) ML algorithms need to be evaluated on out-of-distribution data to assess generalizability, reflective of tumor heterogeneity. (2) Segmentation metrics alone are limited to evaluate the errors made by ML algorithms and their describe their consequences. (3) Adoption of tools in other domains such as robustness (adversarial attacks) and model uncertainty (prediction intervals) lead to a more comprehensive performance evaluation. Such a holistic evaluation framework could shed light on an algorithm's clinical utility and help it evolve into a more clinically valuable tool.

## 1. Introduction

Accurate and consistent segmentation of gliomas (Chen et al., 2017), is important for diagnosis, treatment planning, and post treatment evaluation. Glioblastoma Multiforme (GBM), the most aggressive of high grade gliomas, has the worst prognosis with a 5-year survival rate of <5% and a median survival of approximately a year even with treatment (Tamimi and Juweid, 2017; Witthayanuwat et al., 2018). Low grade gliomas (LGG), though less aggressive than GBM, reportedly undergo anaplastic progression into higher grade tumors around 70% of the time within 5–10 years of diagnosis. The median survival from initial diagnosis is ~7 years (Claus et al., 2015).

Current standard of care for High Grade Glioma (HGG), for example GBM, is surgical resection of the tumor followed by radiotherapy combined with the chemotherapeutic agent temozolomide (Tan et al., 2020). Segmentation for the surgical resection for gliomas should be effective for total gross resection or reduction in tumor bulk, without affecting the surrounding normal functional brain tissue. Radiation therapy requires accurate delineation of tumor margins to ensure effective dosage to tumor region. Due to the relative low aggressiveness of LGG, a more conservative management (“wait-and-watch”) approach (Whittle, 2004) is sometimes adopted. Segmentation is important in this scenario also to monitor temporal morphological and volumetric alterations of the tumors during observation, prior to elective tumor resection (Larsen et al., 2017).

However, the imaging presentation of gliomas varies between LGG and HGG, which could affect the accuracy of their segmentation. Most HGGs, such as GBMs, have a heterogeneous appearance on T1-weighted pre-contrast imaging and typically show a heterogeneous thick-walled rim-enhancing appearance on the T1 post-contrast (T1-Gd) sequence, with a surrounding low attenuation of perifocal edema. The overall appearance of HGGs on T2-weighted fluid-attenuated inversion recovery (FLAIR) sequence is heterogeneously hyperintense, with areas corresponding to enhancing and non-enhancing components as seen on T1-weighted post contrast sequence. The advancing non contrast-enhancing FLAIR hyperintense portions of the tumor are of concern to clinicians because it is believed to contain active tumor remote from the apparent enhanced portions of the aggressive core. On the other hand, low grade tumors appear hyperintense on a FLAIR sequence with or without clear margins. On the pre-contrast T1-weighted sequences, the lesions tend to be hypointense and typically do not enhance following administration of gadolinium based agents (Forst et al., 2014; Bulakbaşı and Paksoy, 2019).

Manually defining the margins of the tumor and surrounding non-enhancing perifocal region remains challenging due to tumor heterogeneity, ill-defined margins, and the varying degrees of perifocal edema. This makes segmentation an arduous task with questionable consistency. In recent years, Machine Learning (ML) techniques have shown potential to assist in tumor segmentation for correct diagnosis and efficient treatment planning (Wadhwa et al., 2019; Bajaj and Chouhan, 2020; Kocher et al., 2020; Nazar et al., 2020). While both HGG, including GBM, and LGG, benefit from accurate segmentation, existing ML validation rarely tests if an algorithm generalizes well to out-of-distribution data that reflects this tumor heterogeneity. Rebsamen et al. (2019) have shown that implicitly incorporating high-vs.-low tumor grade information in model training could improve model performance. While recent work has evaluated for tumor heterogeneity across geographic populations (McKinney et al., 2020), hospital systems (Zech et al., 2018), and federated learning settings (Sheller et al., 2020), this has yet to be done considering differences between HGG, for example GBM and LGG imaging presentations.

**Figure 1:**
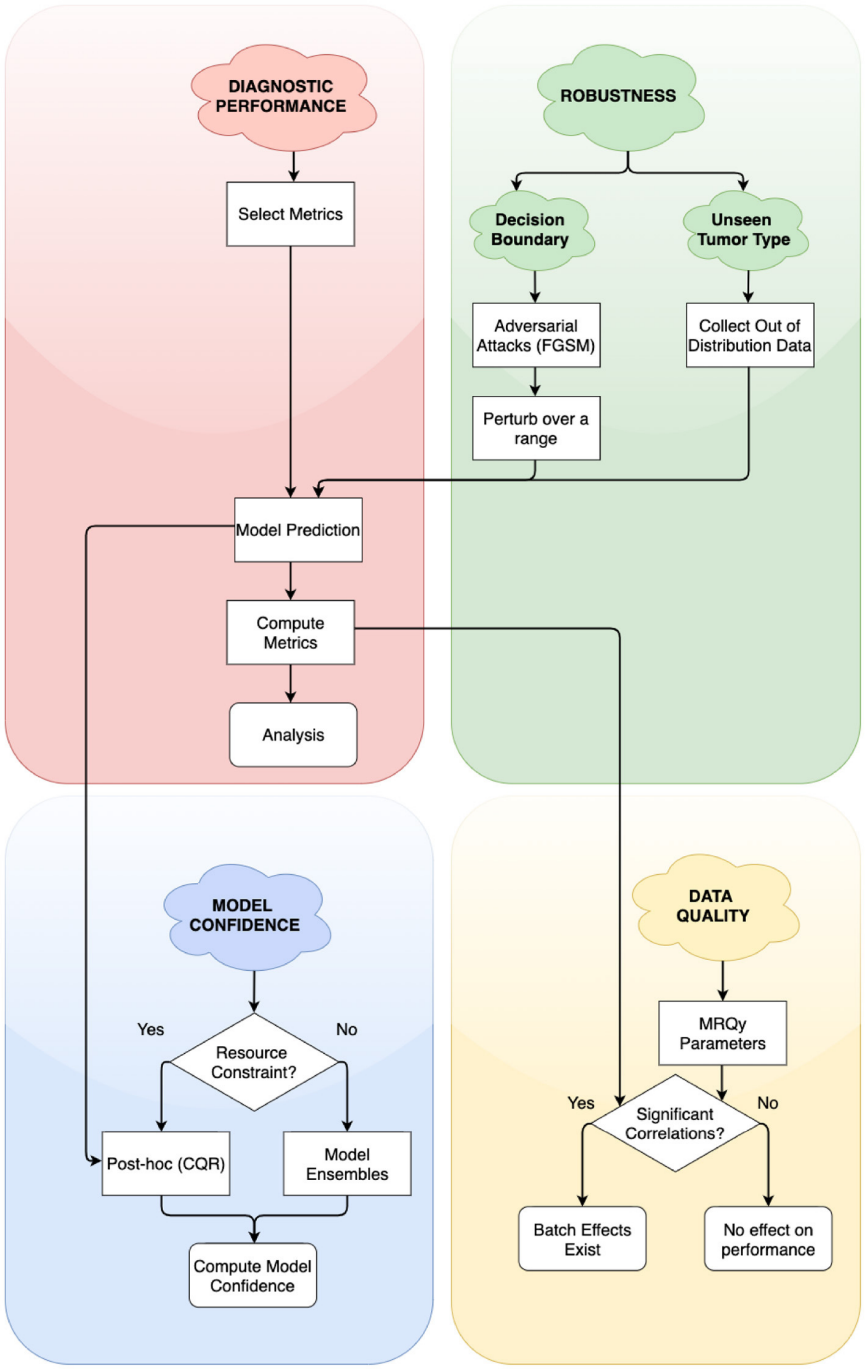
Simplified flowchart of different axes of holistic evaluation—diagnostic performance, robustness, model confidence, and data quality. Axes are ordered by dependency and relation with each other. We recommend models to be evaluated with atleast one experiment on each of these axes. We evaluate two aspects of robustness, namely, closeness to decision boundary and generalizability on unseen glioma type. Decision points in the framework lead to alternate paths for researchers to follow.

In this work, we address the need for rigorous evaluation of ML algorithms for brain tumor segmentation. We propose a holistic evaluation framework (Figure 1) that takes into account tumor heterogeneity, robustness, and confidence of the ML algorithm, and batch effects that may arise from the data. We demonstrate this framework with a cross-sectional study design similar to Zech et al. (2018) and analyze how well an ML algorithm trained on one glioma type (either HGG, exemplified by GBM or LGG) generalizes to another, out-of-distribution glioma type. We conduct four experiments and holistically evaluate an ML algorithm for the problem of tumor segmentation:

**Diagnostic Performance**: We compute standard segmentation metrics to objectively compare the ML algorithm's segmentation performance against radiologist-annotated ground truth. Results indicate that metrics such as Dice and AUROC do not sufficiently capture differences in generalizability, although the classification matrix reveals clear differences.

**Model Confidence**: We measure model confidence in segmentation performance by computing prediction intervals for the brain as well as tumor region. Results indicate that ML algorithms trained on LGG data is more confident than the rest on all homogeneous as well as mixed data.

**Robustness**: We measure the ML algorithm's ability to maintain performance despite adversarial perturbations to test their reliability comparably. Results indicate that the ML algorithm trained only on GBM data was least robust when segmenting tumor corrupted with high levels of noise. Testing performance of the model across out of distribution data, was performed in all the experiments, but can be considered an extension of robustness testing.

**Data Quality (Batch Effects)**: We measure the degree to which MRI scan quality influences segmentation metrics. Results found that scan quality features are not significantly correlated with performance, but that there were some batch effect differences, primarily between LGG and GBM sites.

Our results demonstrate the limitations of segmentation metrics, and caution that metrics alone do not capture all aspects of an ML algorithm's performance. We discuss how our findings relate to recent literature in segmentation metrics. We further discuss how such a holistic evaluation framework could shed light on the algorithm's clinical utility in post-deployment scenarios and help it evolve into a more clinically valuable tool (Recht et al., 2020).

## 2. Materials and Methods

The aim of this work is to propose a framework to evaluate model performance across four axes—diagnostic performance, model confidence, robustness, and data quality. To demonstrate this framework, we first train ML algorithms by considering tumor heterogeneity. We use publicly accessible code for algorithm development and perform *post-hoc* calibration.

### 2.1. Dataset

We used publicly available Magnetic Resonance Imaging (MRI) from The Cancer Genome Atlas (TCGA) (Clark et al., 2013). Glioblastoma Multiforme (GBM) and Low Grade Glioma (LGG) collection (Bakas et al., 2017a,b). This included the skull-stripped and co-registered MICCAI-BraTS 2018 Test Dataset (Menze et al., 2015; Bakas et al., 2017c). The data consisted of pre-operative multimodal MR imaging sequences (i.e., T1, T1-Gd, T2, T2-FLAIR) along with their whole-tumor segmentation labels composed of edema, enhancing tumor, and non-enhancing tumor. We combined these labels into a single whole tumor for this study. Number of patients in GBM BraTS Test Dataset and LGG BraTS Test Dataset were split approximately in half and allotted to validation and test datasets. The GBM and LGG data were merged across the three categories to form an ALL dataset. Each patient was associated with 144 pre-operative MRI scans, which were treated as independent data points for 2D segmentation. These MRI scans were cropped to 144 × 144 pixels and further pre-processed the data by pixel-intensity normalization. Table 1 describes the total number of patients and total number of MRI scans available in each dataset. The training datasets were used for model development (section 2.2), validation datasets were used to determine hyperparameters and calibrate the models (section 2.3), and test datasets (*D*_*GBM*_, *D*_*LGG*_, *D*_*ALL*_) were used to perform subsequent experiments (section 3).

**Table 1:** Split of patients in each of the three datasets.

**Dataset**	**GBM patients**	**LGG patients**	**ALL patients**
Train	102 (14,688)	65 (9,360)	167 (24,048)
Validation	16 (2,304)	21 (3,024)	37 (5,328)
Test	17 (2,448)	22 (3,168)	39 (5,616)

*Values in brackets (.) indicate the total number of images available in the dataset for 2D segmentation. Note that henceforth, we refer to the test dataset as D_GBM_ (GBM patients only), D_LGG_ (LGG patients only), and D_ALL_ (All patients—GBM and LGG patients)*.

### 2.2. Network Architecture and Training

We used the state-of-the-art U-Net architecture (Ronneberger et al., 2015) to develop three tumor segmentation models using the GBM, LGG, and ALL train datasets. The U-Net architecture consists of an encoder, decoder, and skip connections. Each module of the encoder consists of 2D Convolution layers, followed by Batch Normalization and MaxPooling layers. Four such modules make up the encoder. The decoder consists of four modules of Conv2DTranspose layers followed by Concatenate layers. The network performs slice-wise (2D) segmentation with multi-modal MRI scans provided as the input. Models were trained with Dice Loss function for 100 epochs on 8 GPUs. Adam optimizer (Kingma, 2015) was used with a learning rate of 1 × 10^−4^ and a batch size of 128. Data augmentation was used while training each of the models to improve generalization. This consisted of random rotations (0–25° degrees range), random zooming (value = 0.2, zooms image by 80–120% range), width shift (value = 0.2, horizontal translation of images by 0.2 percent), height shift (value = 0.2, vertical translation of images by 0.2 percent), shear (value = 0.2, clips the image in counter-clockwise direction) and random horizontal flips. We referred to publicly available code for model development, model training, and data augmentation (Dong et al., 2017; Ojika et al., 2020).

### 2.3. Model Calibration

The goal of model calibration is to align the algorithm's predicted probabilities align with the observed (ground truth) outcomes (Guo et al., 2017). Calibration process ensure that algorithms do not overstate or understate their confidence in prediction of tumor (Jungo and Reyes, 2019; Mehrtash et al., 2020). Models that have been already trained can be calibrated with *post-hoc* methods (Rousseau et al., 2021). Guo et al. (2017) recommend performing post-hoc calibration with the same validation dataset (Table 1) used for model development. We use Platt Scaling technique (Platt, 1999) for post-hoc calibration due to its simplicity and ease of implementation. To ensure models are properly calibrated, we compute and report common calibration metrics. Negative Log Likelihood (NLL) measures a probabilistic model's quality and is also known as cross-entropy loss. Brier Score (BS) measures the accuracy of probabilistic predictors. Percentage Expected Calibration Error (ECE%) partitions the model's predictions into equally spaced bins and takes a weighted average of the difference between accuracy and model confidence across bins. Percentage maximum calibration error (MCE%) estimates the worst-case deviation between confidence and accuracy. For metric definitions and more information, we refer readers to Mehrtash et al. (2020) and Guo et al. (2017). Table 2 indicates that all models are properly calibrated.

**Table 2:** We first compute calibration metrics on a patient-level, then aggregated by mean.

**Metrics**	***M*_***GBM***_**		***M*_***LGG***_**		***M*_***ALL***_**	
	**Before**	**After**	**Before**	**After**	**Before**	**After**
NLL	0.038212	0.013506	0.070146	**0.022842**	0.056573	0.018483
BS	0.003519	**0.002970**	0.006020	0.005263	0.004533	0.003862
ECE%	0.3413	0.1439	0.5877	0.3141	0.4454	**0.1876**
MCE%	36.4552	14.0762	31.9731	14.3702	37.0614	**13.8812**

*We consider only pixels in the skull-stripped brain to compute these metrics. ECE and MCE are presented in %. Metrics should ideally reduce upon calibration. Columns under each model indicate metric values before and after calibration. Bold values indicate best % decrease or increase as compared to the “before” column. All models improved after calibration*.

## 3. Experiments

Here, we perform an experiment on each of the four axes of our evaluation framework. We compute metrics to summarize diagnostic performance, measure model confidence by computing prediction intervals, simulate adversarial attacks to assess robustness and use MRQy package to analyze batch effects in data. For each experiment, we point to related work, and provide details on the experiment procedure. Then, in section 4, we provide the outcome of these experiments. We evaluate each of the calibrated ML algorithms (*M*_*GBM*_, *M*_*LGG*_, and *M*_*ALL*_) on each of the three test datasets (*D*_*GBM*_, *D*_*LGG*_, and *D*_*ALL*_). Thus, we evaluate 3 (models) × 3 (datasets) = 9 conditions.

### 3.1. Metrics for Segmentation Performance

There exist a plethora of metrics to evaluate the performance of a medical image segmentation algorithm (Udupa et al., 2006; Taha and Hanbury, 2015). Each metric focuses on a specific aspect of the algorithm's performance, and is thus limited in capability to describe the algorithm's performance by itself. Several metrics are necessary to describe comprehensive characteristics of segmentation performance (Renard et al., 2020).

We perform this experiment as a baseline, reflective of the current standard practice for evaluation. We follow the guidelines described by Taha and Hanbury (2015) and select eight metrics to evaluate segmentation performance. Sensitivity (Sens) measures the proportion of tumor pixels that are correctly identified as tumor (foreground). Specificity (Spec) measures the proportion of benign pixels that are correctly identified as benign (background). Positive Predictive Value (PPV) measures the probability that pixels classified as benign truly belong to parts of the patients' brain without a tumor. Negative Predictive Value (NPV) measures the probability that pixels classified as tumor truly belong to parts of the patients' brain with a tumor. While accuracy can be skewed due to the paucity of tumor pixels in the tumor class, Balanced Accuracy (BAcc) takes into account class imbalance. Dice Coefficient (Dice) and Jaccard Coefficient (Jac.C) both measure the overlap between tumor annotated by the different sources (ML algorithm and the radiologists' manual annotations). Area under Receiver Operating Characteristics curve (AUROC) describes the probability that a randomly selected tumor pixel will have a higher predicted probability of being a tumor than a randomly selected benign pixel. We eliminate any extra-cranial regions and only consider the skull-stripped brain for computing the metrics. We compute metrics on a per-patient level, as it offers more granularity than at a population-level.

### 3.2. Prediction Intervals for Model Confidence

Prediction Intervals (PIs) are often reported and considered for medical decision-making (Kümmel et al., 2018). In radiation oncology, Chan et al. (2008) used prediction intervals to capture uncertainty in tumor and organ movement. While a confidence interval measures the precision of a predicted value, PIs measure the expected range where a future observation would fall, given what has already been observed. The width of the PI is directly proportional to the model uncertainty at that region (Kabir et al., 2018). We use prediction intervals to quantify uncertainty in tumor segmentation.

We use Conformal Quantile Regression (CQR) (Romano et al., 2019) to compute PIs. Construction of PIs is difficult, as PIs can be too small that they don't capture the true magnitude (Type 1 error) or too large that they are uninformative (Type 2 error) (Elder et al., 2021). The CQR method guarantees construction of PI such that the target value is contained within the PI by error probability α (valid coverage) and that the PIs are informative.

We used the CQR method to compute PIs in a *post-hoc* manner. The method uses a dataset for training the CQR models and a separate test dataset to compute the PIs. To reduce computational cost, we selected summary images (image with the largest tumor) for each patient in the validation and test datasets (Table 1). We designed a setup to generate prediction intervals around the calibrated model values. We first obtained logits (model output before the calibration) for the selected summary images for patients in both datasets. The CQR models were trained on validation dataset logits and the corresponding calibrated model predictions as target values. The trained CQR models were then used to compute prediction intervals for test dataset logits. We followed the method described by Romano et al. (2019) to compute average prediction intervals (API) per-patient in the test set. We then generated API box plots for all nine conditions.

### 3.3. Adversarial Attacks for Robustness

This experiment was designed to test the impact of data quality and potential batch effects on the predictions of the model. There has been a lot of work in other domains on evaluating the adversarial robustness of ML algorithms. The application of imperceptible noise can change the prediction of image classification system from correctly identifying a panda to confidently miscalling the image a gibbon (Goodfellow et al., 2015). There are now a variety of adversarial attack techniques, from white-box techniques that can look inside the algorithm to those that can build attacks simply by testing inputs and outputs. These techniques can provide a useful framework for evaluating the robustness of a medical imaging machine learning system. In tumor imaging in general, Zwanenburg et al. (2019) showed how radiomics features can be evaluated for robustness by perturbing the tumor mask. Understanding how vulnerable ML algorithms are to noise, and how easily they change their decisions in response, gives a sense of how these ML algorithms might fail.

The adversarial attack used in this experiment was fast gradient signed method (FGSM), described by Goodfellow et al. (2015). This technique is a white-box method which takes the calculated gradient of the neural network to find the direction of the smallest change that will affect the label of the output. This gradient adversarial noise is multiplied by a factor of epsilon, to vary the strength of the attack. In these experiments the epsilon factor was varied over a range of 0–1 (0, 0.005, 0.01, 0.05, 0.1, 0.2, 0.4, 0.6, 0.8, 1.0), with more examples on the lower end of the range to evaluate small perturbations.

We performed the FGSM attack on each of the test datasets (*D*_*GBM*_, *D*_*LGG*_, and *D*_*ALL*_), for all three ML algorithms (*M*_*GBM*_, *M*_*LGG*_, and *M*_*ALL*_). The full panel of metrics was computed for each of these experiments. The performance of the ML algorithms was expected to decay as epsilon decreased, but the relative robustness of each of the ML algorithms and the way that they decayed was studied as well. The chosen epsilon values were (0, 0.005, 0.01, 0.05, 0.1, 0.2, 0.4, 0.6, 0.8, and 1). An epsilon of 0 indicates that no change was made to the image.

### 3.4. MRQy for Analyzing Batch Effects

Magnetic resonance imaging has many strengths in studying and monitoring cancer status, including a variety of sequences to investigate different aspects of tumors. However, the flexibility it provides to radiologists can lead to inconsistencies in protocol and scan quality. MRQy is MRI quality package that provides a variety of features that assess the quality of a scan, and other effects that might be considered batch effects (Sadri et al., 2020). The complexity of machine learning algorithms makes it possible for them to pick up on batch effects between sites rather than the underlying biology of a problem.

These MRQy factors were used to audit the susceptibility of the different ML algorithms to scan quality factors. For each of the MRI sequences, MRQy features were calculated independently on the original NIFTI files. The features used per modality were: MEAN, RNG, VAR, CV, PSNR, SNR1, SNR2, SNR3, SNR4, CNR, CVP, CJV, EFC, TSNEX, TSNEY, UMAPX, UMAPY (For metric definitions, Sadri et al., 2020). The metadata and size features were excluded as they were not available, and the sizing was consistent across all the images. The average true positive probability of a tumor pixel having a tumor label was calculated, as well as for true negative, false positive and false negative pixels. These were calculated on a per patient level and then averaged across all the patients in the test set. These values along with Dice score and AUROC were then assessed for their correlation with the MRQy features using Spearman correlation coefficient. MRQy features that are correlated with model performance are potential quality control metrics that might be used to flag problematic cases. False discovery rate (FDR) correction was then performed using Benjamini-Hochberg correction at an alpha of 0.25 (Benjamini and Hochberg, 1995). We used this correction as it is less stringent than a more aggressive Bonferroni correction and was still found to eliminate the uncorrected *p*-values.

Additionally, independent of the metrics, batch effects were investigated using the MRQy parameters to compare TCGA site codes in the combined testing data set (*D*_*ALL*_). The MRQy features were normalized then decomposed using principal component analysis (Tipping and Bishop, 1999). The first two MRQy principal components and their relationship to institution were investigated using ANOVA and paired *T*-tests in the statsmodels python package (Seabold and Perktold, 2010). We hypothesized that some site differences within the data sets might be captured by this dimensionality reduction.

## 4. Results

In this section, we present and analyze the results of the four experiments in section 3. We discuss their implications in section 6. Note that we perform these experiments for the pixels within the skull-stripped brain.

### 4.1. Metrics Alone Do Not Sufficiently Describe the Nature and Severity of Segmentation Mistakes

**Figure 2:**
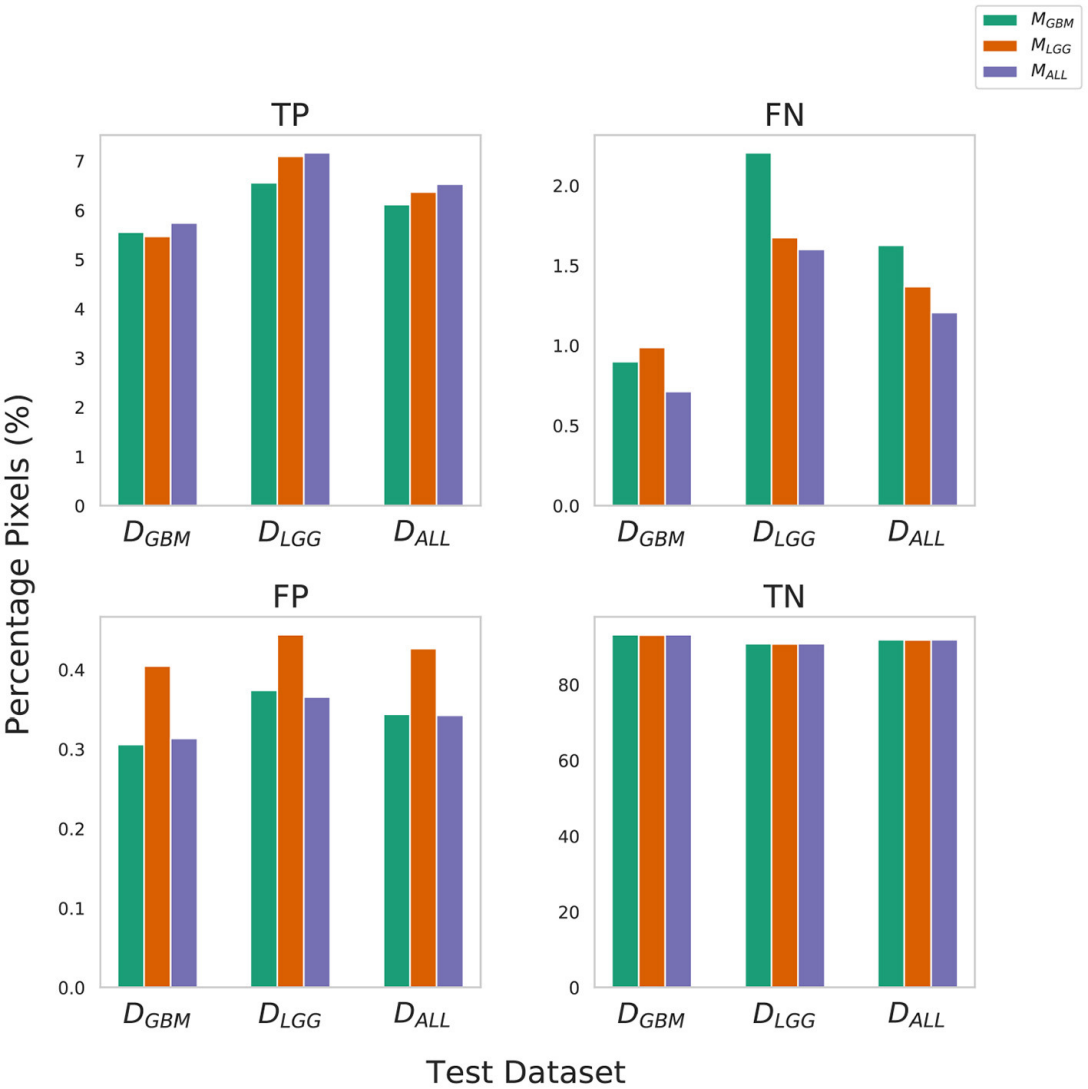
Confusion Matrix to assess the performance of M_*GBM*_, M_*LGG*_, and M_*ALL*_ across stratified and composite datasets. The y-axis denotes percentage of total pixels in a test dataset classified as TP, FN, FP, TN. *M*_*LGG*_ has the tendency to over-segment (high %FP), while *M*_*GBM*_ has the tendency to under-segment tumor(high %FN), relative to each other. Note that metrics such as Dice coefficient routinely ignore the background (TN) in a segmentation context, so a 0.1% difference in false positives should be understood relative to the 6–9% of the volume that is tumor.

True Negative (TN) panel in Figure 2 indicates all models perform equally well in identifying benign pixels. *M*_*ALL*_ has the highest percentage TP, indicating the best performance at correctly identifying tumor pixels. On average, due to a higher percentage of False Negatives than False Positives, all algorithms (*M*_*LGG*_,*M*_*GBM*_,*M*_*ALL*_) under-segment tumor more often than they over-segment. The FP value is highest for *M*_*LGG*_. Thus, out of all models, *M*_*LGG*_ classifies benign regions as tumor the most (over-segments). The FN value is highest for *M*_*GBM*_, on average. *M*_*GBM*_ thus, under-estimates tumor pixels and classifies them as benign (under-segments).

The training of the algorithms further explains these findings. *M*_*LGG*_ learns to pick up subtle patterns in the training phase, and when evaluated on *D*_*GBM*_, classifies normal-appearing tissue as part of a tumor. In contrast, *M*_*GBM*_ is used to seeing dominant contrast patterns, which explains why it misses a lot of tumor pixels in LGG.

**Figure 3:**
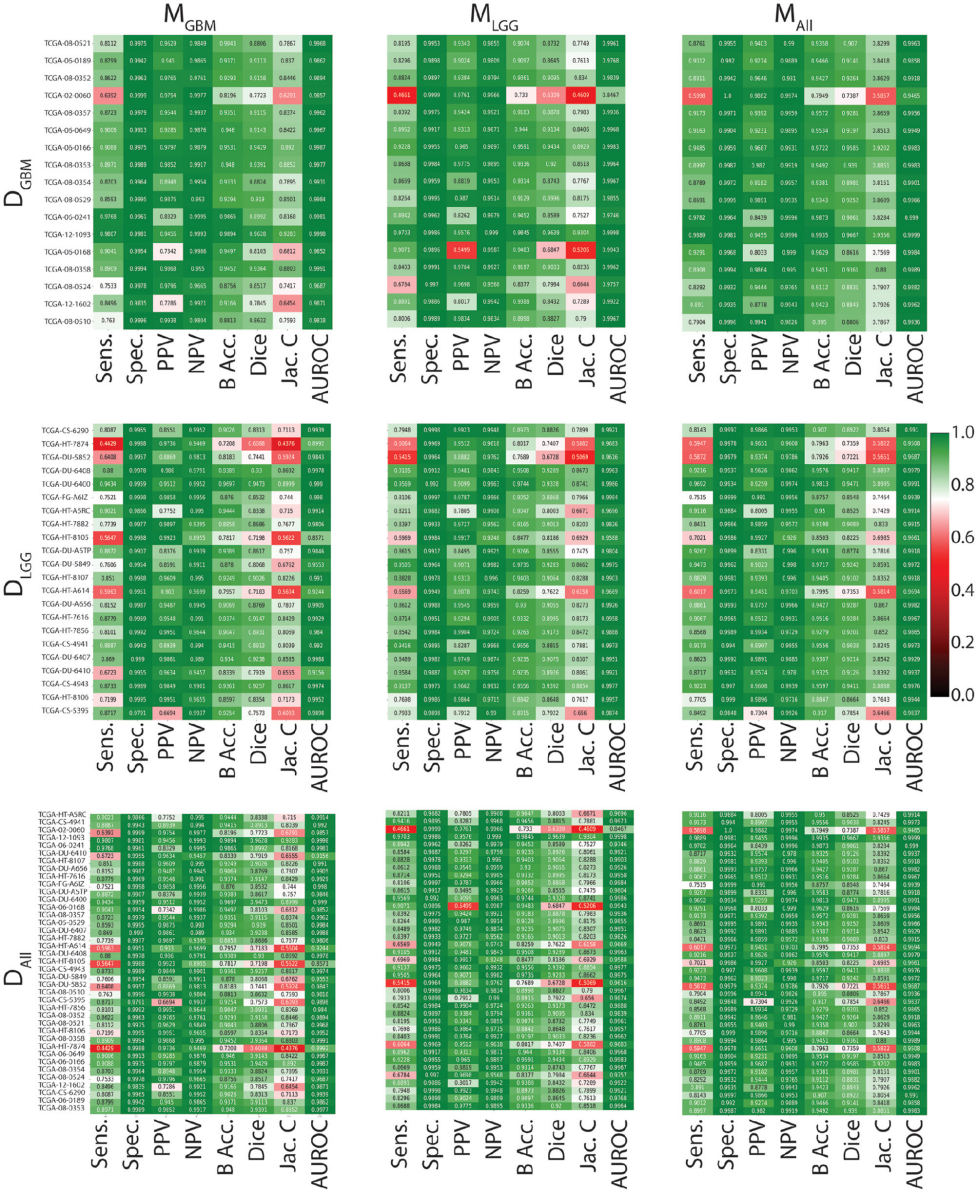
Heat maps indicating patient-level performance metrics. Rows represent test datasets (*D*_*GBM*_, *D*_*LGG*_, *D*_*ALL*_) and columns represent ML algorithms (*M*_*GBM*_, *M*_*LGG*_, *M*_*ALL*_). *D*_*ALL*_ is formed by concatenating the first two rows. In each individual heat map, rows represent model performance on a particular test dataset and columns represent segmentation metrics. Patients for whom all models perform similarly worse are indicated in red.

In Figure 3, all models have similarly worse performance on some patients, indicated by red rows. This is visible across all test datasets. This could be due to multiple confounding variables such as different vendors, field strengths, parameters of imaging, strength of the imaging magnet, type of machine, and it is difficult to pinpoint the contributing factor. Metrics show similar trends in all conditions. Models have a high specificity, low sensitivity, and a high AUROC. There is an overall trend of NPV being higher than PPV. These findings reflect the effect of class imbalance in the dataset, and the models' ability to recognize benign areas much more easily than tumor regions.

### 4.2. Example Illustrations

**Figure 4:**
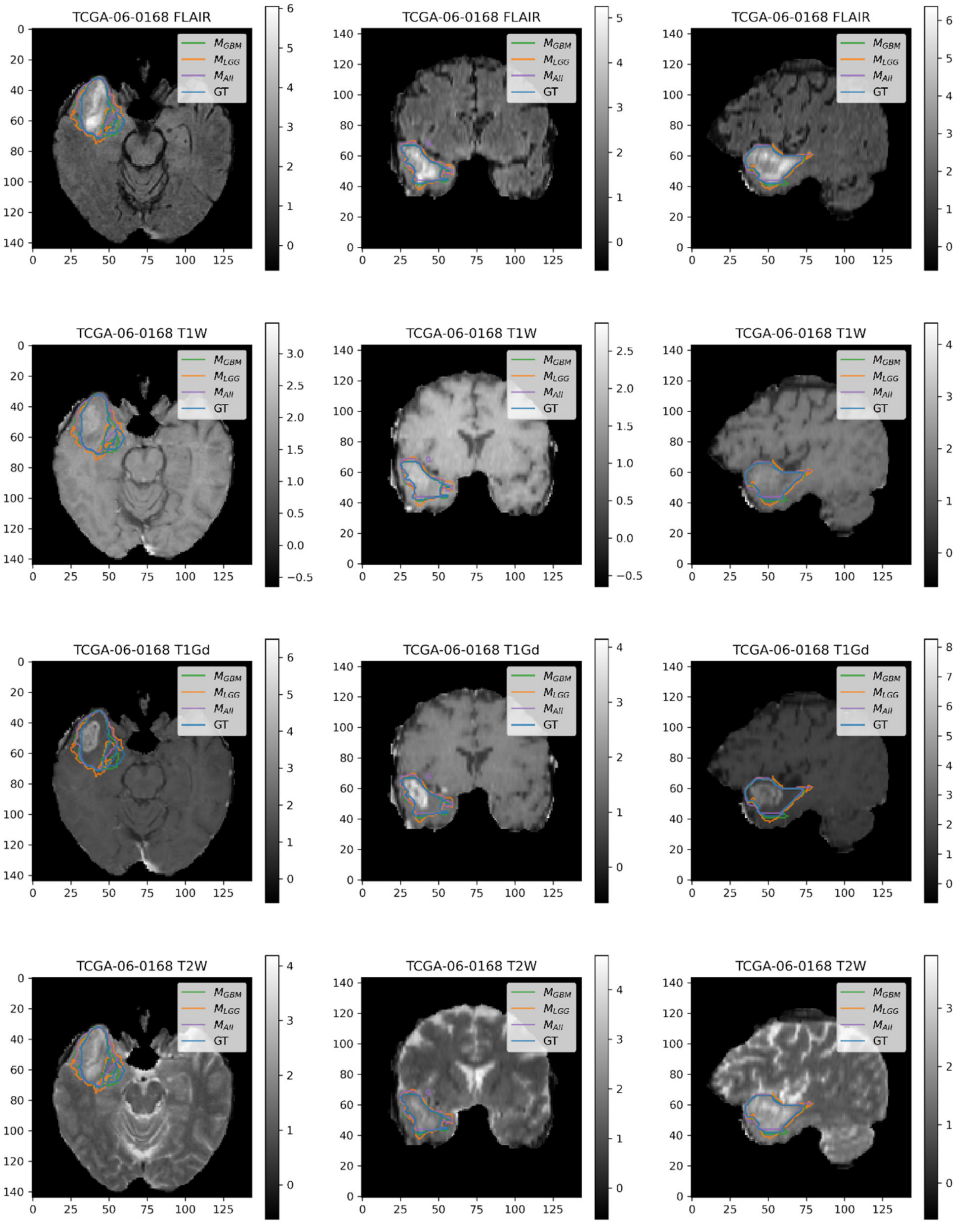
Patient TCGA-06-0168 is diagnosed with GBM in the right temporal operculum. *M*_*LGG*_ has low performance on Dice Coefficient (Dice = 0.6847) than *M*_*GBM*_ (Dice = 0.8103) and *M*_*ALL*_ (Dice = 0.8616). AUROC for all models is high despite unequal performance. The boundary of the edema on FLAIR sequence shows where *M*_*LGG*_ over-segments and *M*_*GBM*_ under-segments tumor.

**Figure 5:**
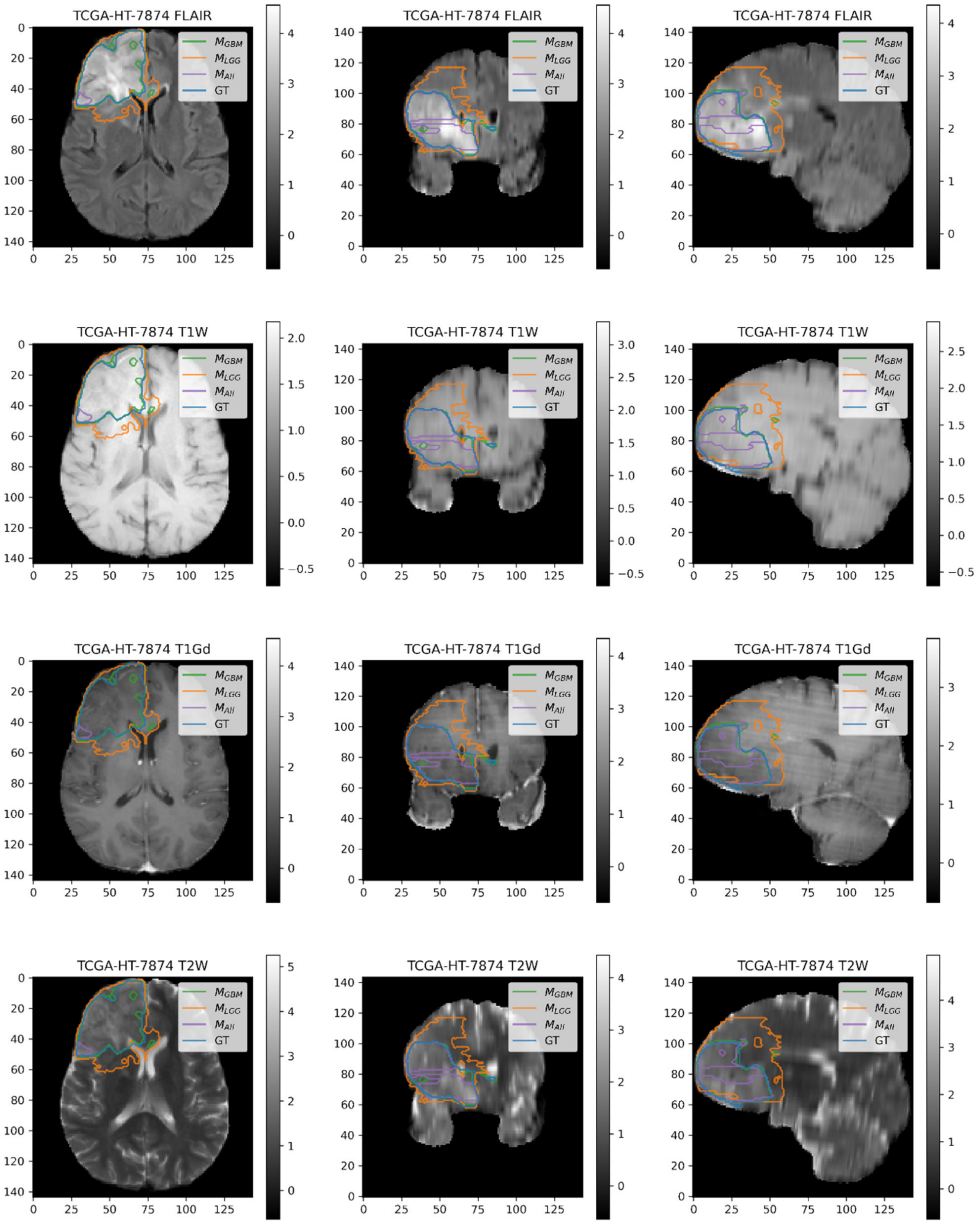
Patient TCGA-HT-7874 belongs to *D*_*LGG*_ and has a tumor in the right frontal lobe. We selected this patient as it has consistently worse performance for metrics (Sens, B.Acc, Dice, Jac.C) across all models. Segmentation plot indicates *M*_*All*_ and *M*_*GBM*_ under-segment in this case, whereas *M*_*LGG*_ over-segments. *M*_*ALL*_ appears to be missing a central part of the tumor, as seen in the coronal and sagittal image planes. *M*_*LGG*_ appears to extend well beyond the region of FLAIR enhancement to over-segment the tumor. This LGG was significantly larger than most LGGs, and that may contribute to the difficulty of segmentation.

**Figure 6:**
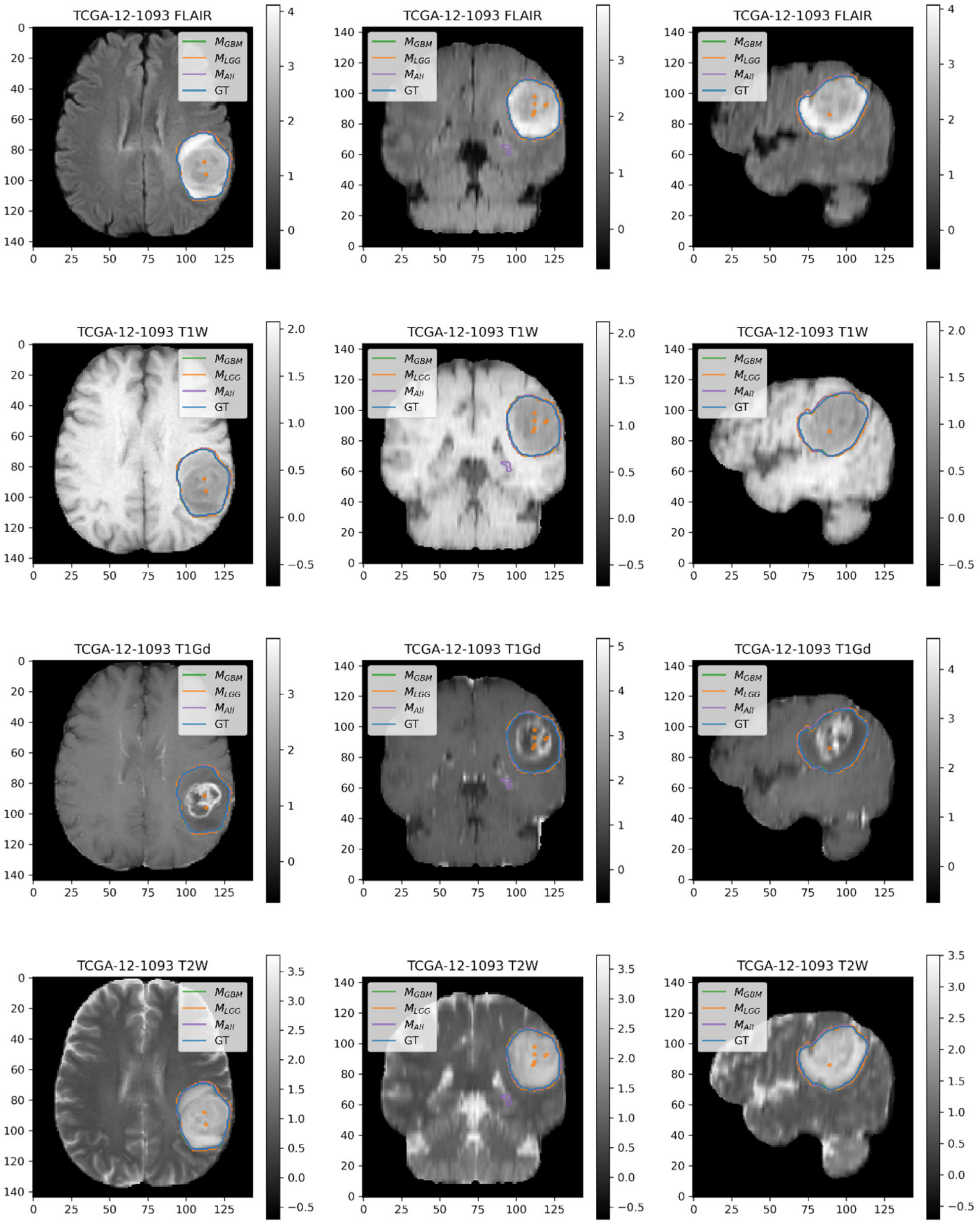
Patient TCGA-12-1093 belongs to *D*_*GBM*_ and has a tumor in the left parietal lobe. We selected this patient as an example because it has consistently good performance for metrics (Sens, B.Acc, Dice, Jac.C) across all models. This GBM has clear margins, and a sharp boundary on FLAIR enhancing regions. The enhancing tumor core is central and distinct, and the models all perform relatively consistently in segmentation.

**Figure 7:**
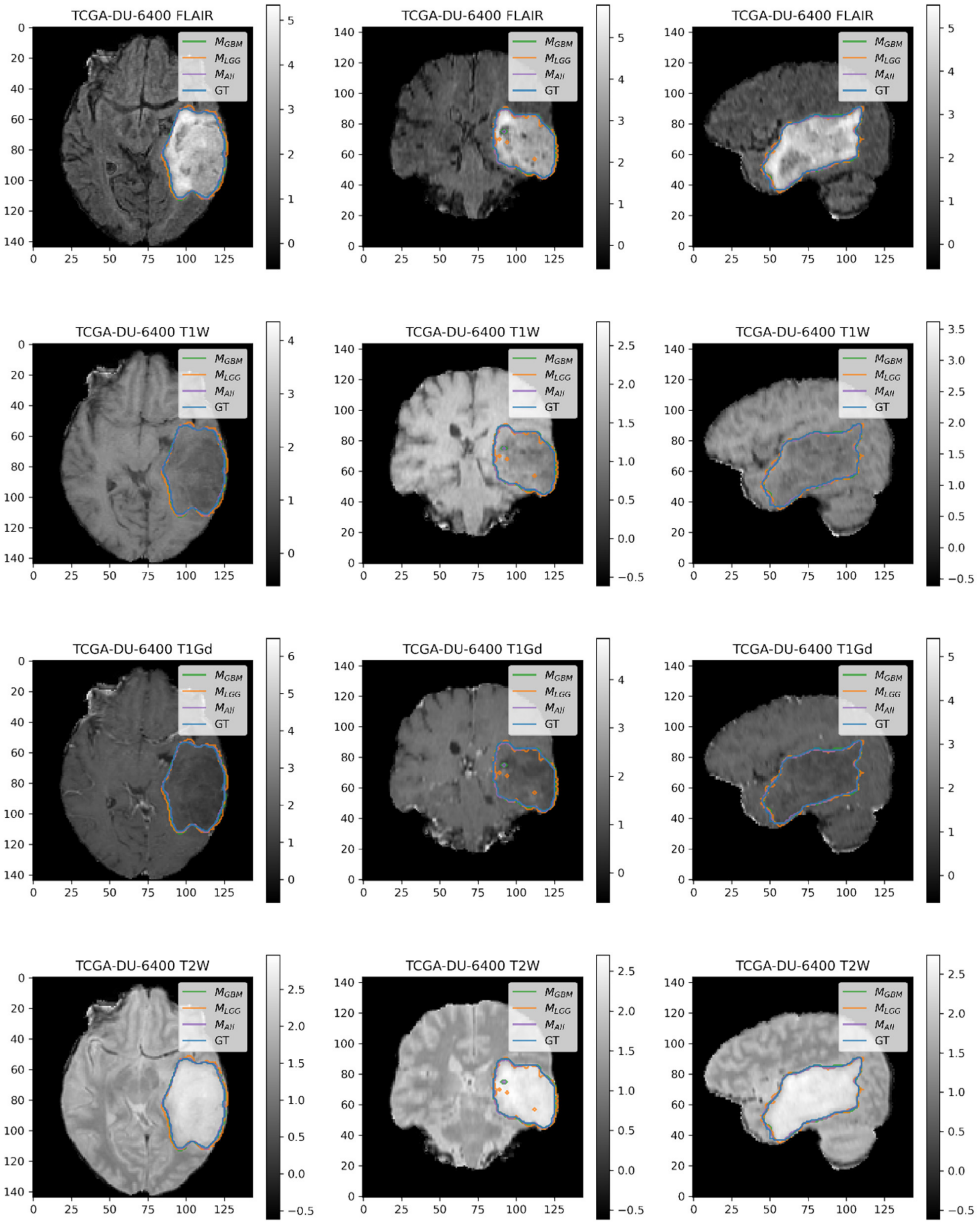
Patient TCGA-DU-6400 belongs to *D*_*LGG*_ and has a tumor in the left temporal parietal region. We selected this patient as an example because it has consistently good performance for metrics (Sens, B.Acc, Dice, Jac.C) across all models. This LGG has clear margins, and the classic signature of FLAIR enhancement and no T1-Gd enhancement.

Here, we present example patients (Figures 4–7) with the Ground Truth (GT) tumor and tumor segmentation contours of *M*_*GBM*_, *M*_*LGG*_, and *M*_*ALL*_. We selected good and bad segmentation examples from *D*_*GBM*_ and *D*_*LGG*_ each for qualitative analysis. One of the authors, who is a board-certified neuroradiologist of more than a decade of experience in brain tumor diagnosis, interpreted these images.

### 4.3. *M*_*LGG*_ Has the Greatest Confidence for Segmentation Across All Datasets

**Figure 8:**
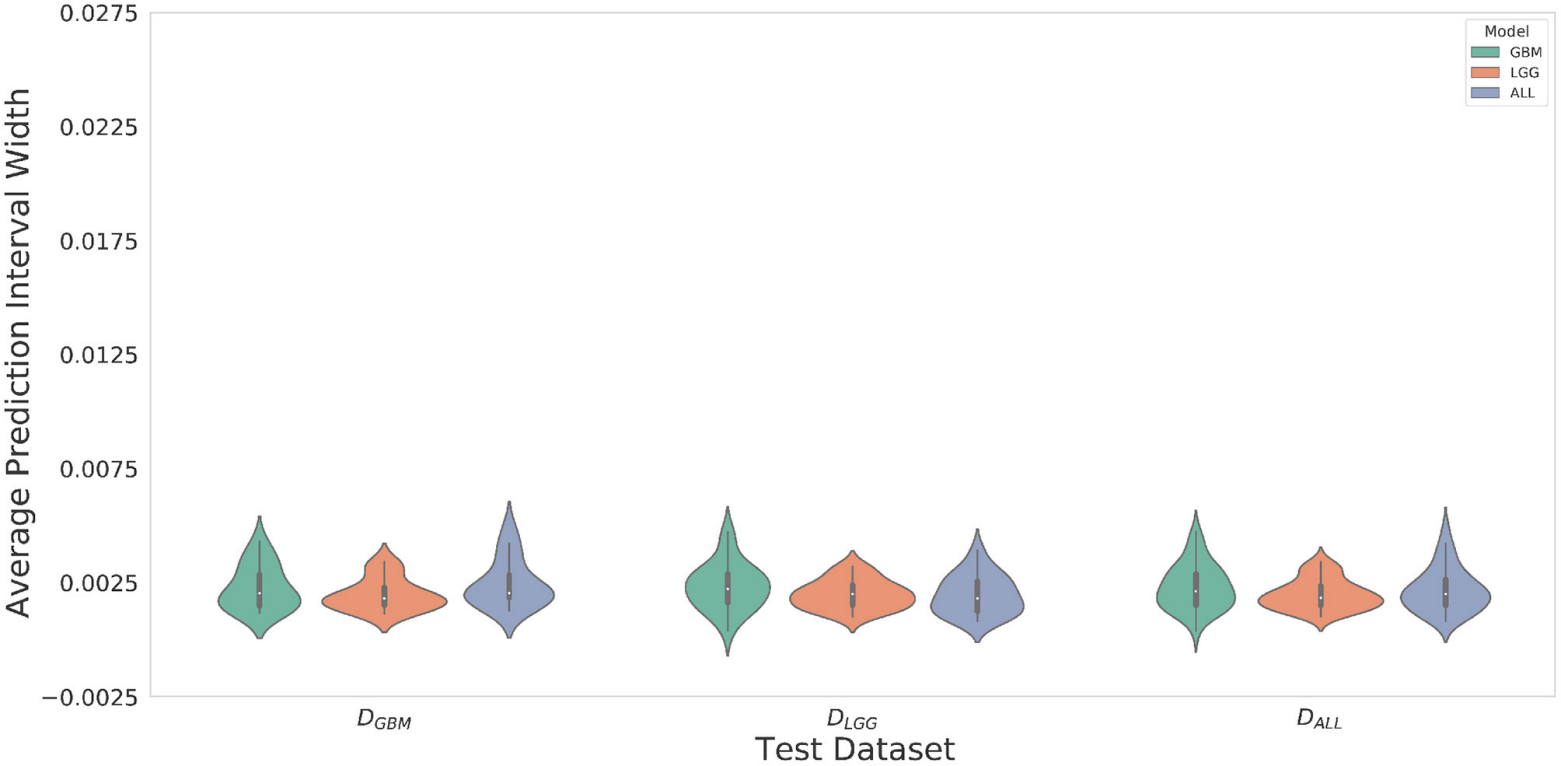
The violin plot indicates models have equal median confidence while segmenting GBM and LGG patients due to greater number of non-tumor pixels in the datasets. The x-axis represents the datasets. The y-axis represents average prediction intervals. Models are sorted by hues and grouped together by test dataset.

**Figure 9:**
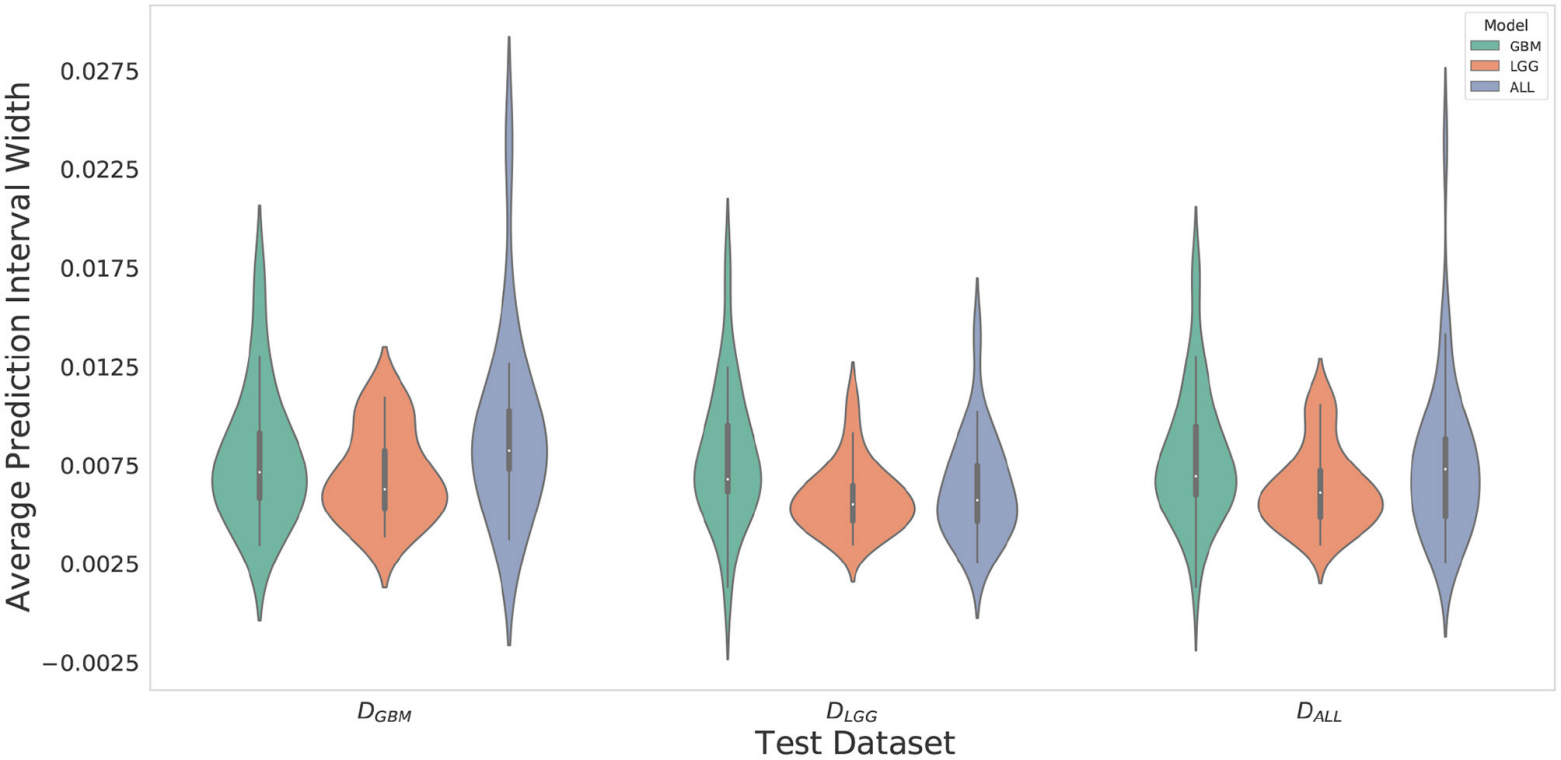
Violin plots constructed to correct for effects of class imbalance and analyze model confidence while identifying tumor pixels only. Plots indicate models confidence is less consistent in identifying tumors due to wider inter-quartile range and greater spread of prediction interval distribution. Plot indicates *M*_*LGG*_ is the most confident model while segmenting both LGG and GBM patients.

Violin plots were constructed to analyze average model confidence across all patients. Figure 8 depicts the average prediction intervals for the skull-stripped brain region. Models have approximately the same median average prediction intervals (API) on each test dataset. Figure 9 represents model confidence while identifying tumor regions. Models have wider inter-quartile range and greater variability compared to Figure 8. This indicates models have low confidence in identifying tumors as compared to non-tumor. *M*_*GBM*_ and *M*_*ALL*_ have similar distributions of API across patients, indicating both models are similarly confident while segmenting both GBM and LGG tumor. *M*_*LGG*_ has the lowest median prediction interval widths, and their distribution has the lowest variability and highest concordance. This indicates *M*_*LGG*_ is the most confident model while segmenting both LGG and GBM patients. Out of all models, *M*_*LGG*_ is consistently confident while making predictions.

*M*_*LGG*_ has the highest confidence, even though it makes mistakes (over-segments) in segmentation, suggestive of an aggressive approach. *M*_*GBM*_ also makes mistakes (under-segments) but has lower confidence, which suggests a cautious approach. LGG may be monitored for a longer period of time, so a high rate of false positives can overburden clinicians, going against the goal of reducing their burden. If mistakes are very obvious, it can cause a high degree of frustration and eventual abandonment of the algorithm (Beede et al., 2020). Previous works have proposed monitoring cases with low confidence (Kompa et al., 2021). However, in a case where a model makes mistakes with high confidence, a confidence-based screening approach might cause the reviewer to miss important areas of model failure.

### 4.4. Models Trained on *D*_*GBM*_ Deteriorated the Most Under Adversarial Attacks

**Figure 10:**
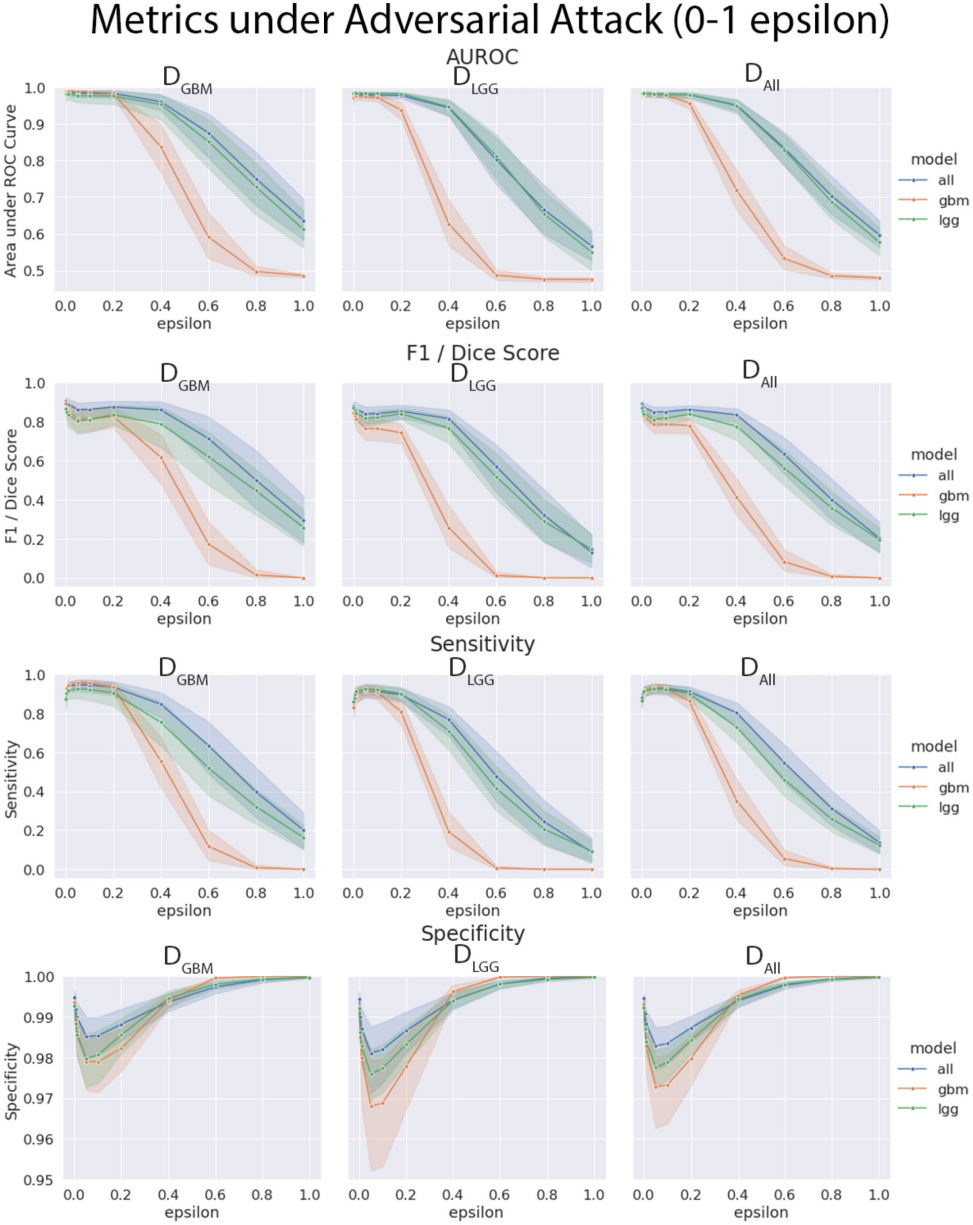
Robustness of each model under FGSM attack, across the full range of epsilons (0–1.0) for four selected metrics. Ninety-five percent confidence intervals are provided to each model, and each of the three data sets were evaluated. *M*_*GBM*_ was least robust to FGSM attack at higher epsilon values with regard to AUROC, Dice score, and sensitivity.

**Figure 11:**
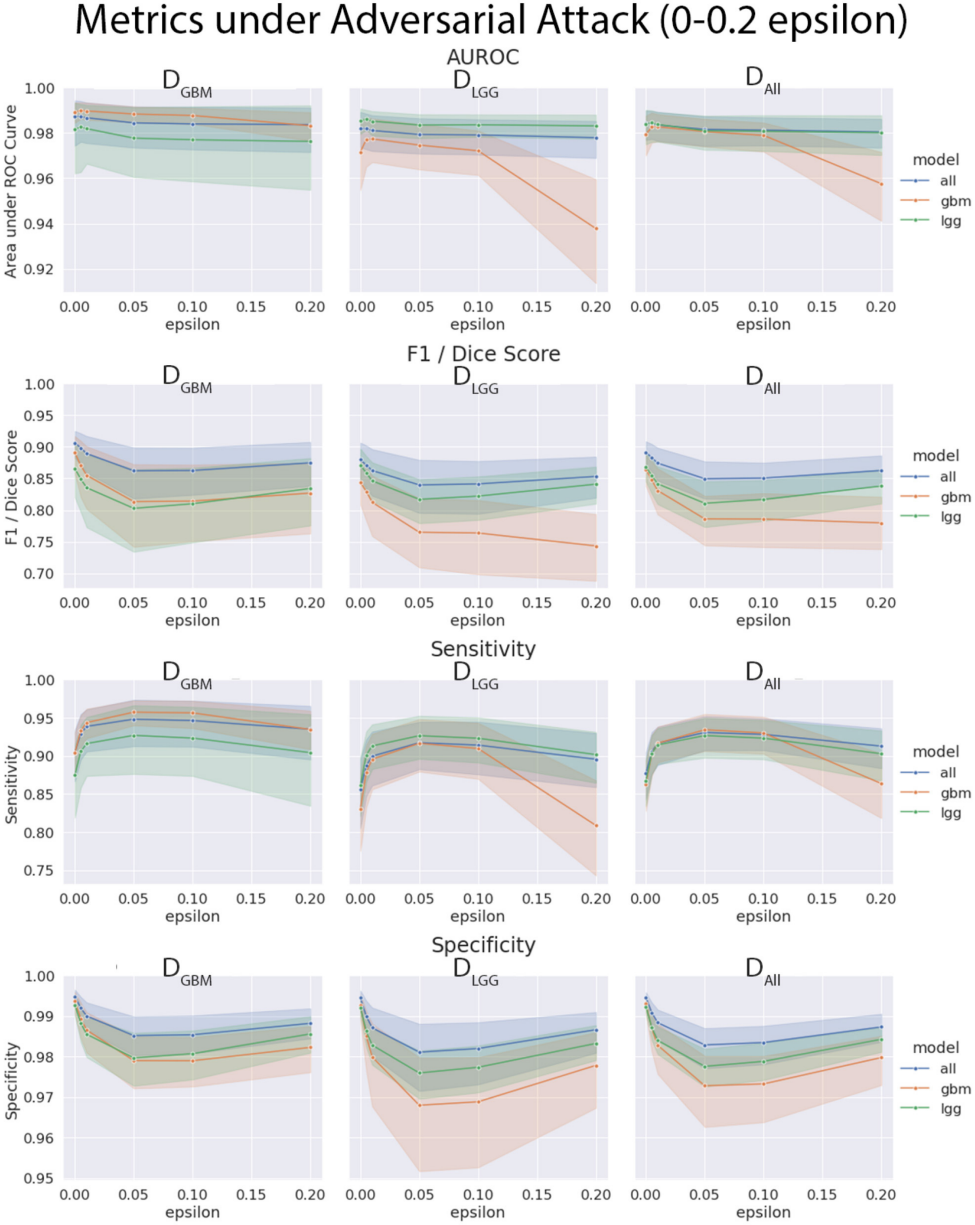
Robustness of each model under FGSM attack, zoomed in on the early range of epsilons (0–0.2) for four selected metrics. Ninety-five percent confidence intervals are provided to each model, and each of the three data sets were evaluated. Models had more similar performance in the less aggressive levels of attack, with all model having marginally better performance, except with *M*_*LGG*_ and *M*_*GBM*_ models performing better with AUROC on their own test data sets.

The three models (*M*_*GBM*_, *M*_*LGG*_, *M*_*ALL*_) were each evaluated on the three test datasets under FGSM attack across a range of epsilons from 0 to 1. The 95% confidence intervals are also included for each of the metrics that were evaluated on a per patient level. *M*_*GBM*_ was the least robust to this type of FGSM attack, across all three test datasets for AUROC, Dice score, and Sensitivity. This might be due to the somewhat consistent imaging presentation of glioblastomas. It was marginally more robust to attack on its own datatype (*D*_*GBM*_). All three models failed by losing sensitivity instead of specificity, indicating that the models began drastically under-segmenting the tumor under high levels of noise. Figure 10 highlights the model behavior under different levels of noise. Under smaller amounts of noise (Figure 11), the all model had the best performance generally, though not significantly. *M*_*LGG*_ and *M*_*GBM*_ had the highest AUROC values of the three models for *D*_*LGG*_ and, *D*_*GBM*_ respectively, though the differences did not reach the significance threshold of (*p* < 0.05).

We found that the models trained only on *D*_*GBM*_ were less robust to adversarial noise, particularly at high levels of adversarial noise. These levels of noise may be extreme, but do give some sense of the performance of the models under duress. Other types of attacks that might be worthwhile to investigate include: adversarial patch attacks, Carlini and Wagner attacks, projected gradient descent, as well as GAN based attacks (Carlini and Wagner, 2017; Brown et al., 2018; Ren et al., 2020). This is not the only way of assessing robustness of models, as it assumes a motivated attacker to guide attacks, as opposed to natural sources of error, but it addresses how the margins of the tumor are affected on a consistent scale across the models. Natural sources of error are less coherent, comparable, and not as well computationally modeled in MRI as the body of work on adversarial attacks.

### 4.5. MRQy Features Vary Between Data Sets and Institutions, but Are Not Significantly Correlated With Metrics

The calibrated models' metrics and probabilities were evaluated for correlations with MRQy parameters, across the different test datasets. While there were some limited parameters that had significant correlations with model metrics, this was before FDR correction. One Thousand two hundred and twenty-four parameter to metric comparisons (17 MRQy parameters, 4 sequences, 6 metrics, 3 models) were performed, and none of the parameter-metric pairs were significantly correlated after FDR correction (*p* < 0.05). The MRQy features were collected before preprocessing, and were shown to be different across different institutions. However, the model used preprocessed data, and the MRQy features were not significantly correlated with the models' predictions and performance. This negative result adds more confidence to the predictions of the machine learning pipeline.

**Figure 12:**
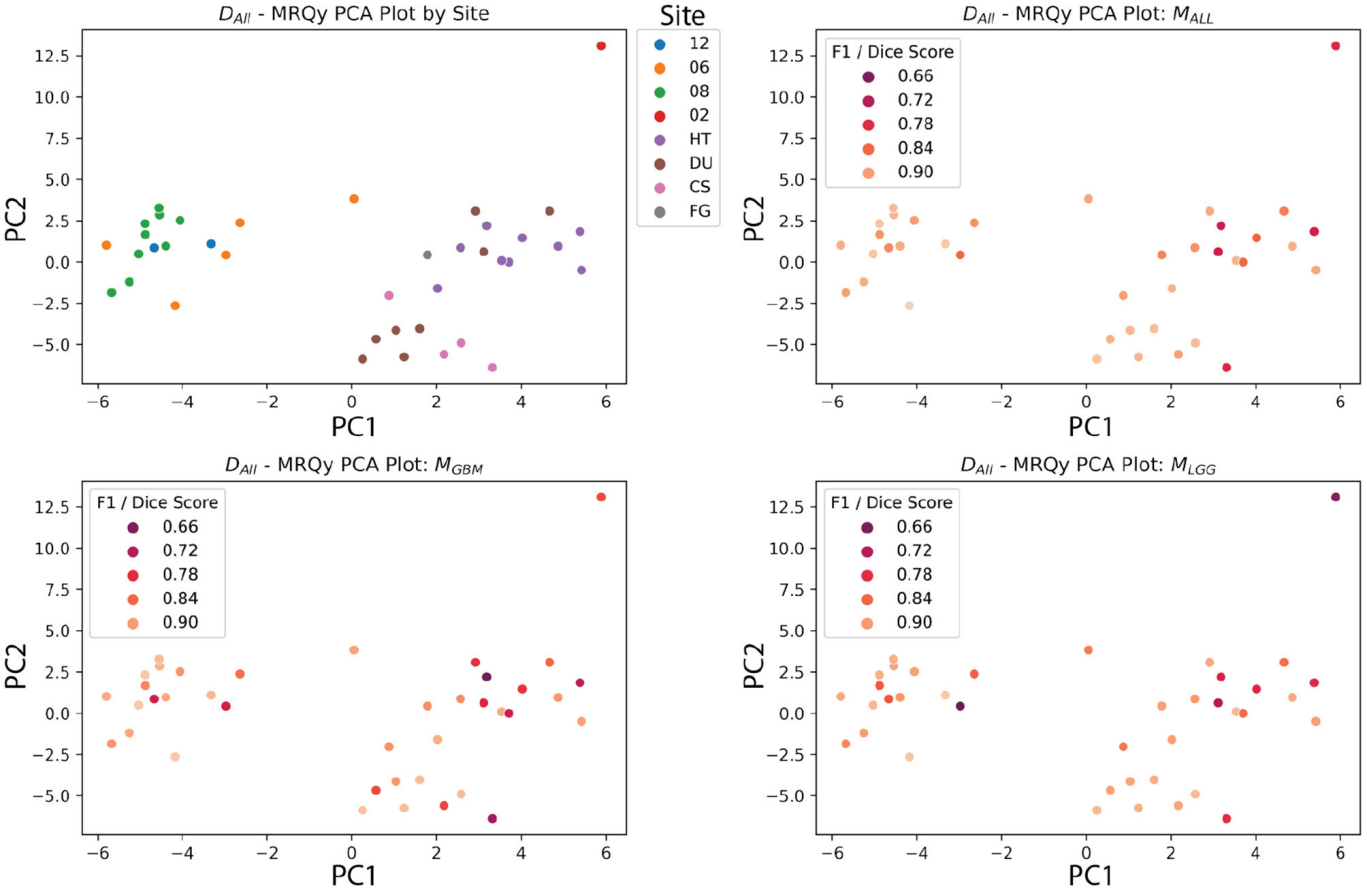
MRQy features after principal component analysis, plotted against site code, and Dice scores of three models. The *D*_*ALL*_ test dataset has the first two principal components of the MRQy features plotted. Pairwise *t*-tests found that three clusters had significant differences in PCA space (06, 08, 12), (HT, DU, CS, and FG), and (02). Notably, these numeric codes happen to correspond to GBM studies, and the letter codes happen to correspond to LGG studies.

The PCA analysis showed that there were significant differences between three groups of site codes. The first cluster of institutions was 12, 06, and 08, the second was HT, DU, CS and FG, and the last was 02. Paired *t*-tests showed that the first principal component created splits with significant differences (*p* < 0.05). Notably, the numerical codes (02, 06, 08, 12) correspond to GBM studies, and alpha codes corresponded to LGG studies (HT, DU, CS, FG). However, within these clusters, the differences didn't reach significance. Figure 12 shows the site codes plotted in PCA space, and then the three models with Dice coefficient. The fact that Henry Ford Hospital (06 for GBM and DU for LGG) had more in common with other GBM and LGG sites than between those two groups is notable, though hard to explain with such a limited sample size. Site 02 was also an outlier from both other clusters in this PCA space, and had relatively poor performance, though with one case it's hard to draw a firm conclusion.

The BraTS 2018 test datasets (Menze et al., 2015) did not have significant correlations after FDR correction between scan quality and metrics. This could be due to the high-fidelity curation and good consistency of the dataset. Another potential explanation could be the limited size of the dataset. Still, these data quality metrics show significant correlations with TCGA sites after PCA analysis, indicating batch effect differences, at least between the GBM and LGG datasets. Other data quality issues that models should be tested for include bias based on race, sex, and socioeconomic status. The rise of federated learning models makes this more urgent, because they allow for training models across collaborators without sharing data (Kairouz et al., 2021). Since sensitive data is not shared between sites, tracking batch effects and sources of bias requires more work and planning than if all the data were shared and managed centrally.

## 5. Discussion

In this work, we used publicly available data and compared three U-Net-based algorithms in a stratified manner. Our main finding is that traditional segmentation performance metrics do not capture all aspects of an algorithm's performance, and can be potentially misleading. In this section, we first discuss the limitations of segmentation metrics, and how our proposed evaluation framework leads to a better understanding of model performance. We discuss the four axes of evaluation—diagnostic performance, model confidence, robustness, and analysis of batch effects in detail. Finally, we address the practical utility of our framework and list recommendations for model evaluation.

### 5.1. Limitations of Segmentation Metrics

Despite the technological advancements of Machine Learning (ML), the adoption of Ml in clinical workflows remains limited (Caruana et al., 2015; Strickland, 2019; Beede et al., 2020). This divide between the development and adoption of ML algorithms has been termed the “translation gap” (Steiner et al., 2021). This limitation is in part due to lack of holistic evaluation of the performance of those ML systems.

Majority of existing algorithms are statistically validated only using segmentation metrics (van Kempen et al., 2021), such as Dice Coefficient (Dice, 1945). In our experiments, we followed guidelines (Taha and Hanbury, 2015) to compute several segmentation metrics and test the differences between segmentation of GBM and LGG patients. We hypothesized that segmentation of LGG patients would be more difficult than GBM patients. LGG is diffuse and has low proliferation, which makes accurate segmentation of submicroscopic tumor tissues and tendrils, a difficult task. In contrast, GBM has greater signal intensity and characteristic presence of necrotic cavities, which makes segmentation comparatively more obvious. Our results found that metrics alone were insufficient to highlight the severity of mistakes that models make in segmentation. Only when segmentation contours were interpreted by a board-certified neuroradiologist, the degree, and types of errors of these models were evident. Similarly, in a recent systematic review of glioma segmentation algorithms, van Kempen et al. (2021) expected to find performance differences in segmentation of HGGs and LGGs but found that reported metrics could not capture such differences.

This points to a bigger concern raised by Reinke et al. (2021) that metrics alone are insufficient to evaluate all aspects of segmentation performance. While metrics are important for objective performance evaluation, they have several limitations for clinical utility (Maier-Hein et al., 2018). Difference in consequences of an algorithm's errors cannot be uncovered by metrics alone, and requires a clinical expert to elucidate them. For example, the consequences of under-segmenting in *D*_*GBM*_ might be more severe than under-segmenting in *D*_*LGG*_ due to the prognosis and management of the two diseases. As LGGs may merit a more conservatory, “wait-and-watch” approach, tumor that might be previously missed can be caught with additional tests. However, segmentation in case of GBM has more immediate consequences for resection and radiotherapy. Under-segmentation in this case would result in non-total resection, and perhaps if tumor tissue remains, would increase the likelihood of recurrence. Over-segmentation on the other hand would cause removal of non-tumor regions of the brain, or subject them to higher levels of radiotherapy, potentially causing functional impairments for patients. In case of glioma, the Dice Coefficient has a limited utility for evaluation of multifocal lesions (Giannopoulos and Kyritsis, 2010) because it cannot represent over-segmentation and under-segmentation (Yeghiazaryan and Voiculescu, 2018), does not support segmentation of multiple structures (Yeghiazaryan and Voiculescu, 2018), and is not immune to imaging artifacts and shape differences (Reinke et al., 2021). This serves as a cautionary tale that metrics alone are insufficient for reporting model performance, and there is clearly a need for better evaluation and reporting standards (Nagendran et al., 2020).

Since medical data is tightly controlled to protect patient privacy, federated learning has risen as a methodology to train models without exposing data. However, while the cross-site training structure has it's advantages, it requires thoughtful planning of model evaluation since model designers will not have access to the underlying data from other sites. Any metrics, quality control features, and batch effect monitoring will have to be carefully pre-planned to judge any resulting models. Thorough and holistic evaluation is especially important as site variability in protocol and patient populations is a known confounding factor. Our framework also helps illuminate the axes on which a federated learning network should judge their models beyond simple metrics like accuracy or AUROC.

### 5.2. Dimensions of the Evaluation Framework

The goal of our work is to inform how researchers can holistically evaluate their segmentation algorithms, and consider other axes of model performance than metrics alone. A problem faced by model developers in this domain is the lack of large datasets to effectively train and evaluate their algorithms. To realistically recreate this, we worked with smaller test datasets from TCGA-GBM and TCGA-LGG. Our work explores the effects of working with limited data, and informs how to interpret results meaningfully in such scenarios. Our experiments and methodology stand independently of whether the model evaluator has pre-built models, or is yet to train them. Our framework considers tumor heterogeneity, limitations of metrics and evaluates other axes such as model confidence, robustness, and batch effects. We don't suggest completely abandoning metrics—they would be important as a start, to get some level of insight. However, we caution against solely relying on metrics, and propose a more holistic evaluation of algorithms. In Figure 1, we map the axes of evaluation onto the standard ML pipeline. We provide other potential experiments that researchers can choose for model evaluation along specific axes. For example, techniques such as model ensembles and k-fold cross validation can be used to evaluate model confidence.

In our experiments, we evaluate model robustness with adversarial attacks. Recent work has shown the importance to evaluate the models' abilities to withstand adversarial attacks, especially in high-stakes scenarios such as radiology (Wetstein et al., 2020). These attacks can arise due to strong financial interests or technical infrastructure. We designed this experiment to test how and in what way could models fail in deployment under such an attack. This could lead to appropriate safeguards being put in place. Adversarial attacks also help shed light on the decision boundary of a neural network (Woods et al., 2019), which is otherwise something of a black box. Other sources of noise could be added, but have their own complications. Adding Gaussian noise to the inputs can be difficult to calibrate and variable due to randomness. Addition of artifacts, such as motion artifacts, is complex to model, and tools for doing so are not publicly available. Further research should investigate models using these failure modes, but is outside the scope of this paper. Another axes we investigate is analyzing the dataset for batch effects. In the context of tumor segmentation, batch effects could occur when image acquisition parameters or technical variations correlate with measurement quantity (Sadri et al., 2020). This may become a major problem when it leads to incorrect conclusions (Leek et al., 2010), especially when ML algorithms learn to pick up on these patterns. Analyzing for batch effects thus becomes important, as model predictions can be correlated with confounding factors. Our experiments found that pre-processing might help in making MRI scans more homogeneous and reduce these correlations.

We demonstrated our evaluation framework on ML algorithms trained with reliable, high-fidelity. expert-annotated BraTS Datasets. To further simplify the process of model development, we used straightforward implementations such as fixed dataset split (testing/validation) and 2D segmentation to work with limited data. Model developers can certainly use more sophisticated techniques that result in higher accuracy. Despite these limitations, our experiments are aligned to the overall goal of this work. Another limitation is we consider LGG for evaluation of generalizability. While there are significant imaging differences as compared to GBM, LGG is a broad category consisting of a range of tumor types. A more clinically useful investigation would be to evaluate performance on WHO recognized genetic subtypes such as IDH-mutant vs IDH-wt or 1p/19q codeleted tumors, as the literature on tumor subtypes evolves (Louis et al., 2016). However, we defer this as future work.

### 5.3. Recommendations for Evaluation of Tumor Segmentation Algorithms

Here, we summarize our work and presented the following recommendations for holistic evaluation of ML algorithms:

**Accounting for tumor heterogeneity in evaluation:** We focus on a specific problem of glioma, and evaluate for differences in models trained by stratification of GBM and LGG Data. The first stage in standard of care for glioma is the identification of the type, which further dictates the prognosis and treatment planning. However, there is high variability in this stage, and experts often don't reach immediate consensus. It is thus important for ML algorithms to generalize well across all tumor grades. We set out to investigate this question, by performing holistic evaluation on LGG, GBM, and mixed data. Researchers should consider unique imaging presentations of each patient and evaluate on a patient-level, as important differences might be diminished upon aggregation of data. Researchers should avoid evaluation on a dataset-level.

**Adoption of tools in other domains to investigate glioma segmentation:** Domains such as adversarial robustness and statistics have highly specialized tools (e.g., FGSM, conformal prediction intervals) to interrogate different aspects of model performance. In this work, we demonstrate the value of adopting such tools for the problem of performance evaluation of glioma segmentation. Our results indicate clear differences in these experiments. We found model trained on LGG Data to be more confident, and model trained on GBM to suffer the most under adversarial attacks. Researchers should evaluate their algorithms on each of the evaluation axes, by performing at least one experiment on each of the axes (Figure 1).

**Exploring limitations of metrics in clinical utility:** In recent years, the community has started to acknowledge the clinical limitations of standard segmentation metrics. Our work demonstrates why evaluation by metrics alone is limiting in investigating heterogeneity in clinical populations (i.e., GBM vs. LGG patients), and our findings further support recent literature. Researchers should avoid relying solely on metrics to evaluate their models.

The framework can further shed light on the practical utility of an algorithm, and serve as a decision-support tool. It is not meant to replace the triaging mechanisms already in place. Since the action that accompanies a decision is different, researchers should know the situations and the patient case before use of these algorithms. If the algorithm's prediction would be followed by a high-stakes action component such as surgery, tumor resection, or radiation therapy, accuracy of segmentation is critical. Our results indicate that algorithms trained on a specific glioma grade group do not generalize well out of distribution, so it is best to use specifically-trained models. For example, if a patient with GBM is to undergo surgery, use of *M*_*GBM*_ as a decision-support tool would be best. In low-stakes scenarios such as accessing the extent of tumor infiltration, generalizability is more important at the cost of accuracy. The use of *M*_*ALL*_, which has knowledge of all glioma grade groups, would be best in this scenario.

Establishing a close collaboration with a clinical expert is crucial to ensure that results of the framework are appropriately interpreted. In this work, the authors collaborated with experts in neuroradiology and radiation oncology to deep-dive into the problem of brain tumor segmentation and present the limitations of metrics in a clinically meaningful way. Researchers should similarly consult a clinical expert to understand how tumor heterogeneity manifests in imaging presentations between the subgroups of the tumor they are interested to investigate. The use of this framework in other domains would thus require a close collaboration between ML researchers and clinicians for effective investigation.

## 6. Conclusion

In this work, we proposed a framework to evaluate the performance of tumor segmentation algorithms. To illustrate the framework, we investigated the generalizability of algorithms in different glioma grade groups. Institutions such as the American College of Radiology, Data Science Institute (ACR DSI) often lay out guidelines to researchers for best practices before model deployment. However, it is often not clear to researchers on how to evaluate models. We take a more granular view and present a tutorial of sorts, in addition to proposing a holistic framework for better model evaluation. In addition, we provide the following recommendations to researchers: (1) Perform at least one experiment on model confidence, diagnostic performance, data quality and robustness. (2) Perform analysis on a per-patient basis. (3) Gather representative images informed by the results of such analysis. (4) Collaborate with a clinical expert to perform qualitative evaluation of these images to get deeper insight on model performance.

## Data Availability Statement

Publicly available datasets were analyzed in this study. This data can be found here: https://www.med.upenn.edu/sbia/brats2018/data.html.

## Author Contributions

NCW: data acquisition and pre-processing. VA, NCW, and SP: design of the experiments. SP and NCW: performing experiments and data analysis. AR, NCW, and SP: results interpretation. SP, NCW, NB, and XH: writing of the manuscript. AR: conception and design of study project and supervision. NB and XH: co-advising. JRB: clinical interpretation and guidance. All authors contributed to the article and approved the submitted version.

## Funding

SP was supported by UM-MICDE Catalyst Grant (to XH and AR). AR was supported by CCSG Bioinformatics Shared Resource 5 P30 CA046592, a Research Scholar Grant from the American Cancer Society (RSG-16-005-01), and a Precision health Investigator award from U-M Precision Health to AR (along with L. Rozek and M. Sartor). SP and AR were also partially supported by the NCI Grant R37-CA214955. AR, NCW, XH, and NB were also supported by the University of Michigan (U-M) startup institutional research funds.

## Conflict of Interest

NCW was a founder and shareholder of Prenovo, EIQ, and AMI healthcare technology startups. AR has a consulting agreement with Voxel analytics LLC. and consults for Genophyll, LLC. The remaining authors declare that the research was conducted in the absence of any commercial or financial relationships that could be construed as a potential conflict of interest.

## Publisher's Note

All claims expressed in this article are solely those of the authors and do not necessarily represent those of their affiliated organizations, or those of the publisher, the editors and the reviewers. Any product that may be evaluated in this article, or claim that may be made by its manufacturer, is not guaranteed or endorsed by the publisher.
