# Supplementary material for: Stratification by Tumor Grade Groups in a Holistic Evaluation of Machine Learning for Brain Tumor Segmentation
Source: Front Neurosci. 2021 Oct 6;15:740353. doi: 10.3389/fnins.2021.740353 (PMC8526730; doi:10.3389/fnins.2021.740353)
Supplement: Supplementary file 1 [file Data_Sheet_1.PDF]

frontiers

Supplementary Material

1 SUPPLEMENTARY DETAILS : MODEL CALIBRATION

Here we outline the procedure for Platt Scaling. We first trained a logistic regression model with parameters $a, b \in \mathcal{R}$, optimized using negative log likelihood as loss function. It outputs calibrated probabilities $q_i = \sigma(az_i + b)$, where z_i are the logits, or the network's. Logits are used to obtain the network's predicted probability as $p_i = \sigma(z_i)$. For Platt Scaling, we first obtain z_i by passing X_{val} through the model and extracting the logits. Then, we use y_{val} as y_i. We then fit a logistic regression model on this dataset. Then we find the parameters a, b using $\{z_i, y_i\}$. For prediction, $q_i = \sigma(az_i + b)$. For this, we first obtain logits of X_{train} from the model and consider that as z_i. We then use the parameters of the trained logistic regression model (a, b) to transform logits to calibrated probabilities.

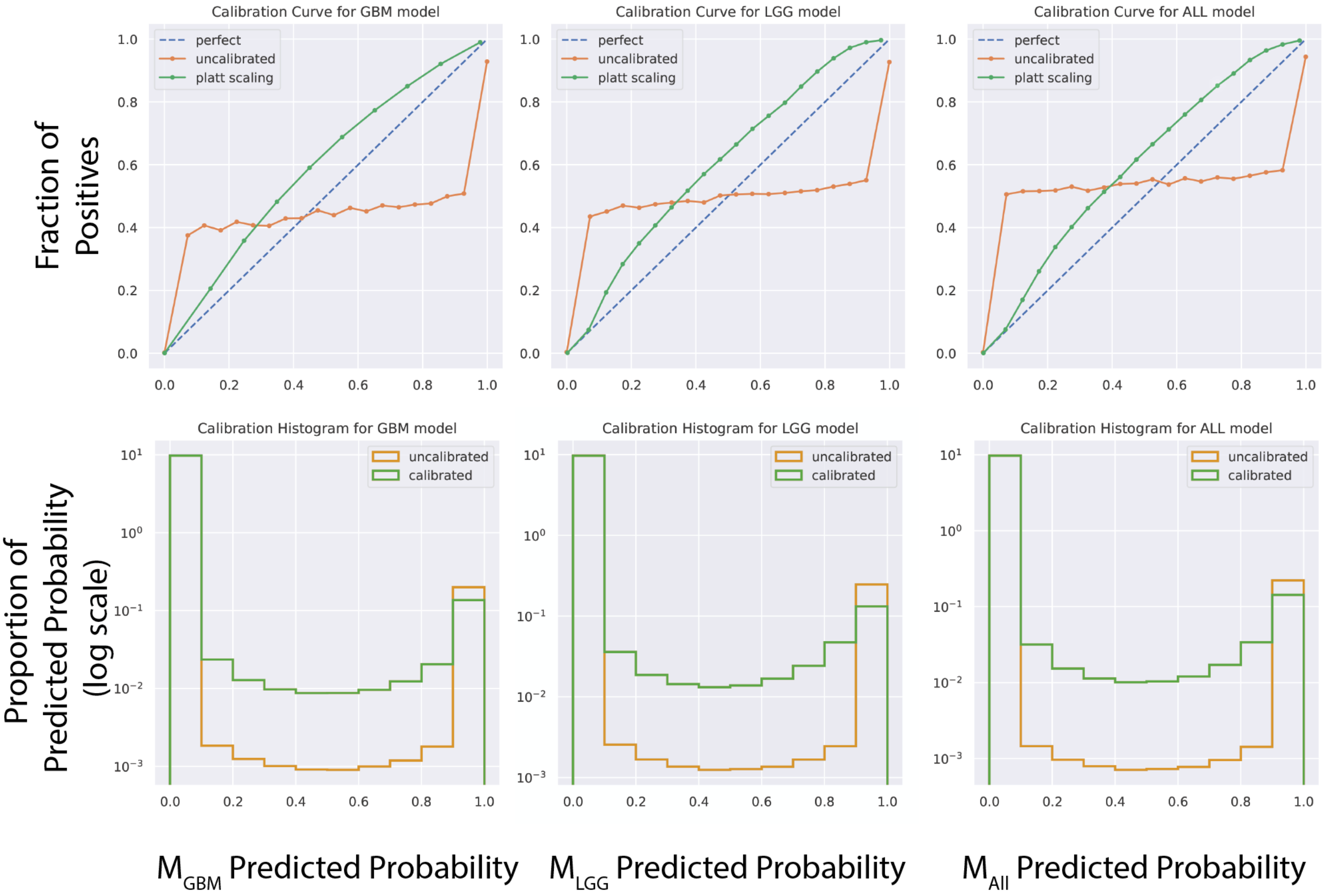

Figure S1. Calibration plots for all models. Calibration is performed post-hoc. Plots indicate that calibration process aligns model predictions to closely reflect the ground truth distribution. Histograms indicate calibration results in a more even distribution of pixels.

2 SUPPLEMENTARY FIGURES: DIAGNOSTIC PERFORMANCE

Here, we present additional images (TCGA-02-0060)from GBM Test Dataset and (TCGA-DU-6410) from LGG Test Dataset.

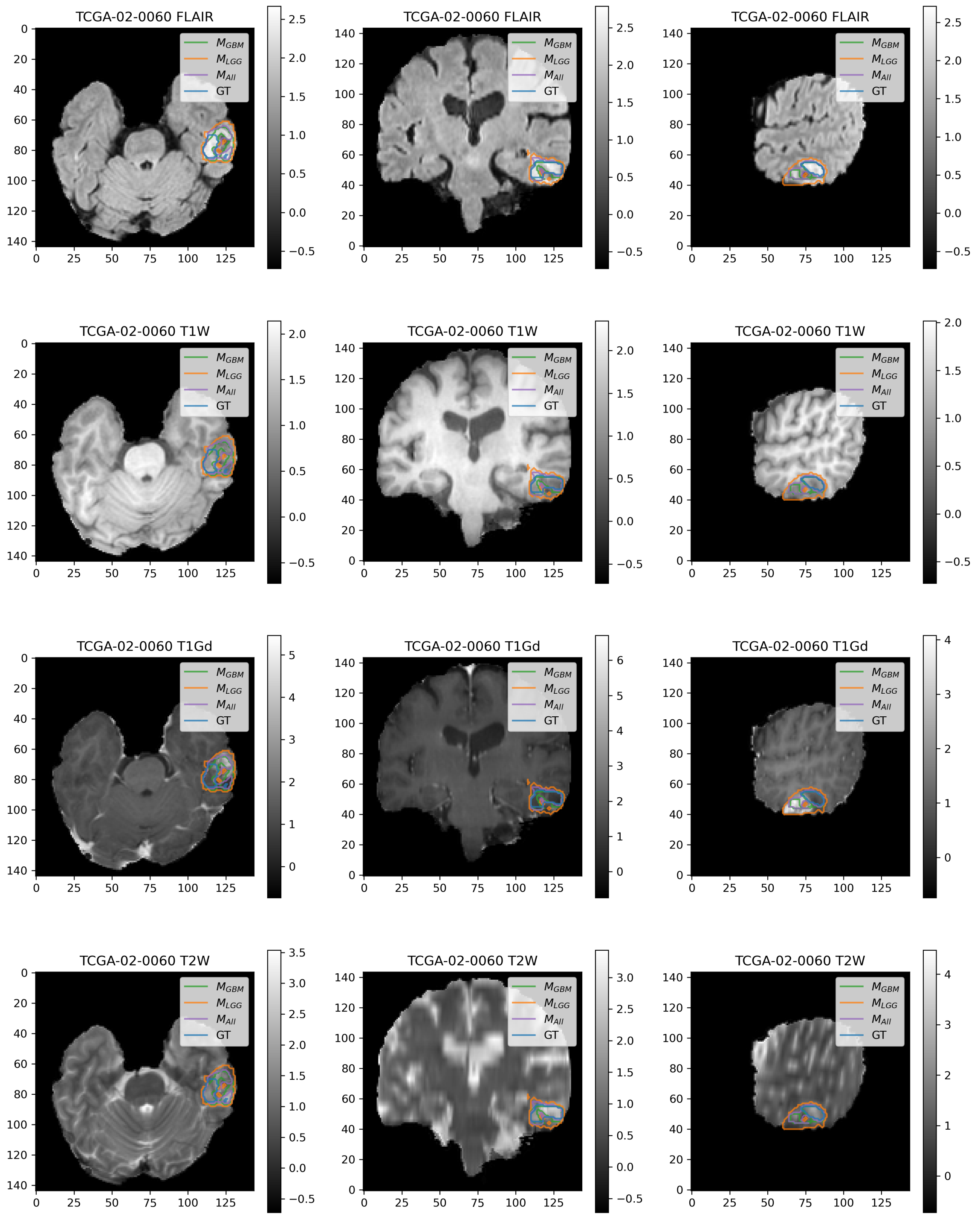

Figure S2. Patient TCGA-02-0060 belongs to D_{GBM}. M_{GBM}, M_{LGG} and M_{ALL} has low performance on multiple metrics, including Dice Coefficient (M_{GBM} Dice:0.7723 , M_{LGG} Dice:0.6309 and M_{ALL} Dice:0.7387). M_{LGG} indicates over-segmentation.

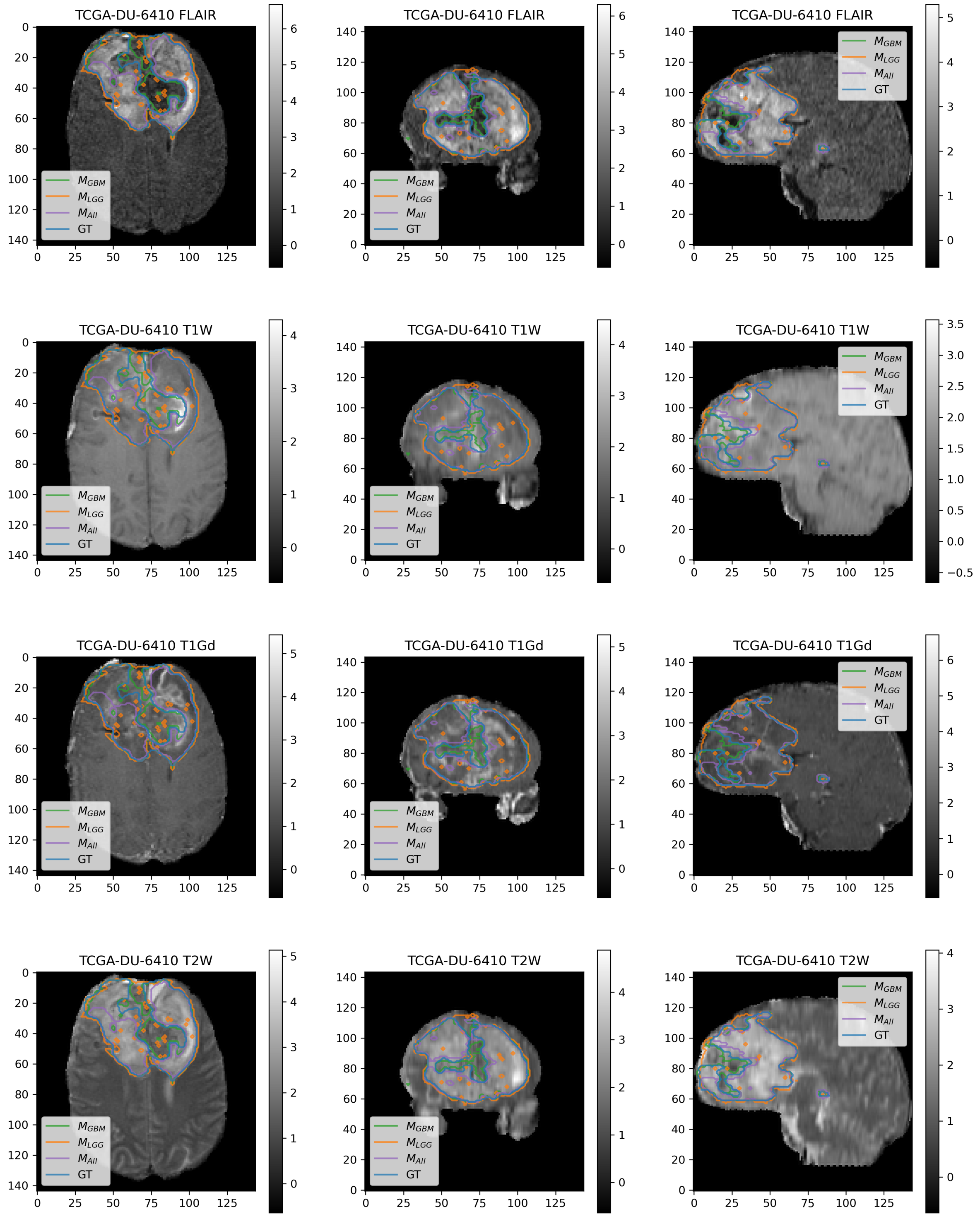

Figure S3. Patient TCGA-DU-6410 belongs to D_{LGG}. M_{GBM} has low performance on Dice Coefficient (Dice = 0.7919) while other models have higher performance (M_{LGG} Dice:0.8926 , M_{ALL} Dice:0.9126). Plots indicate M_{GBM} under-segments the tumor region

3 SUPPLEMENTARY FIGURES: METRICS

Here, we present 8x8 pair plots of metrics used to evaluate diagnostic performance of models.

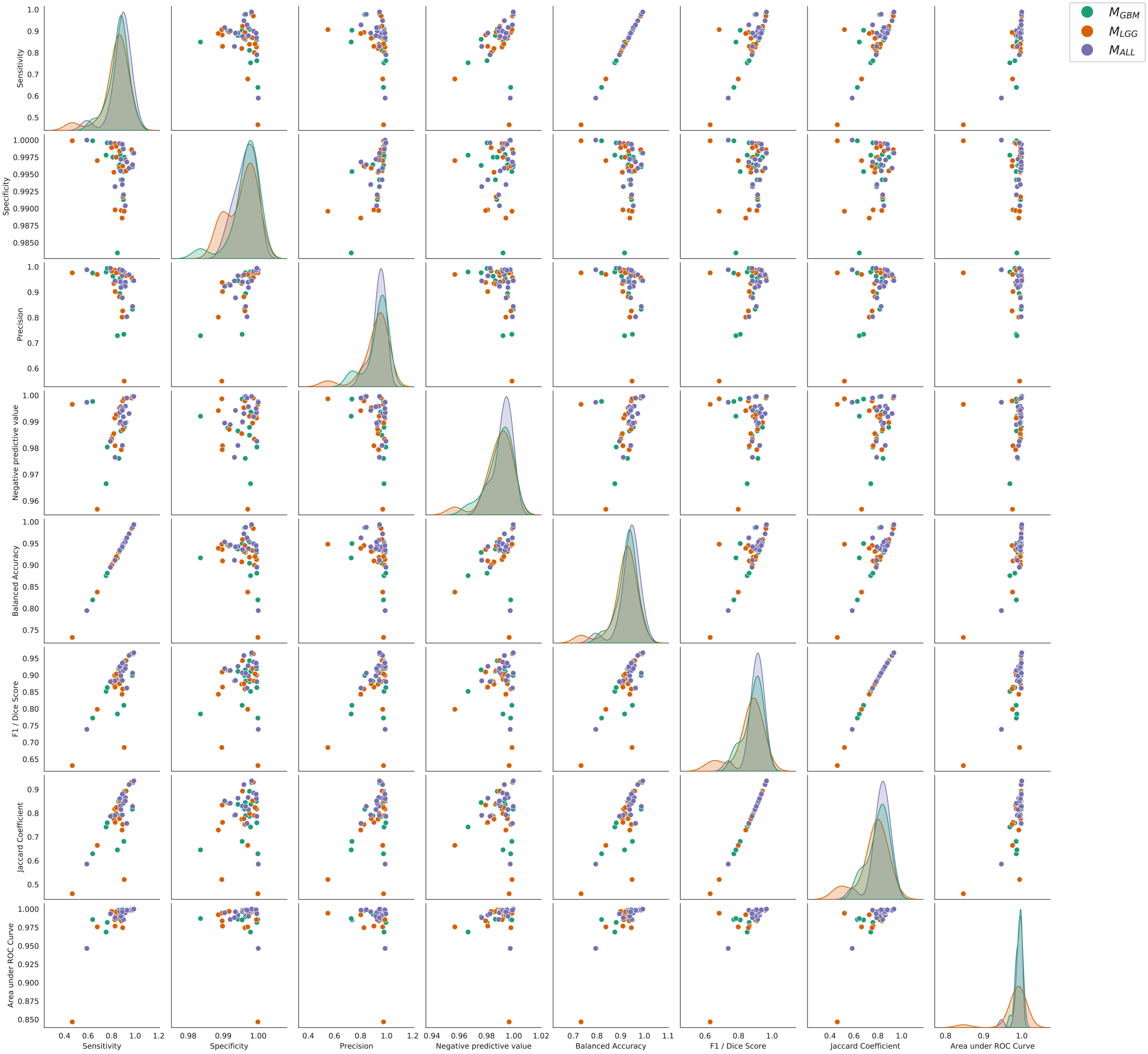

Figure S4. Pair plot of segmentation metrics of each patient in D_{GBM} across three ML algorithms. Marginal plots along the diagonal represent distributions for each metric across three ML algorithms. Dot plots on the off-diagonal represents the two-dimensional relationships between each pair of metrics. ML algorithms are annotated by colors.

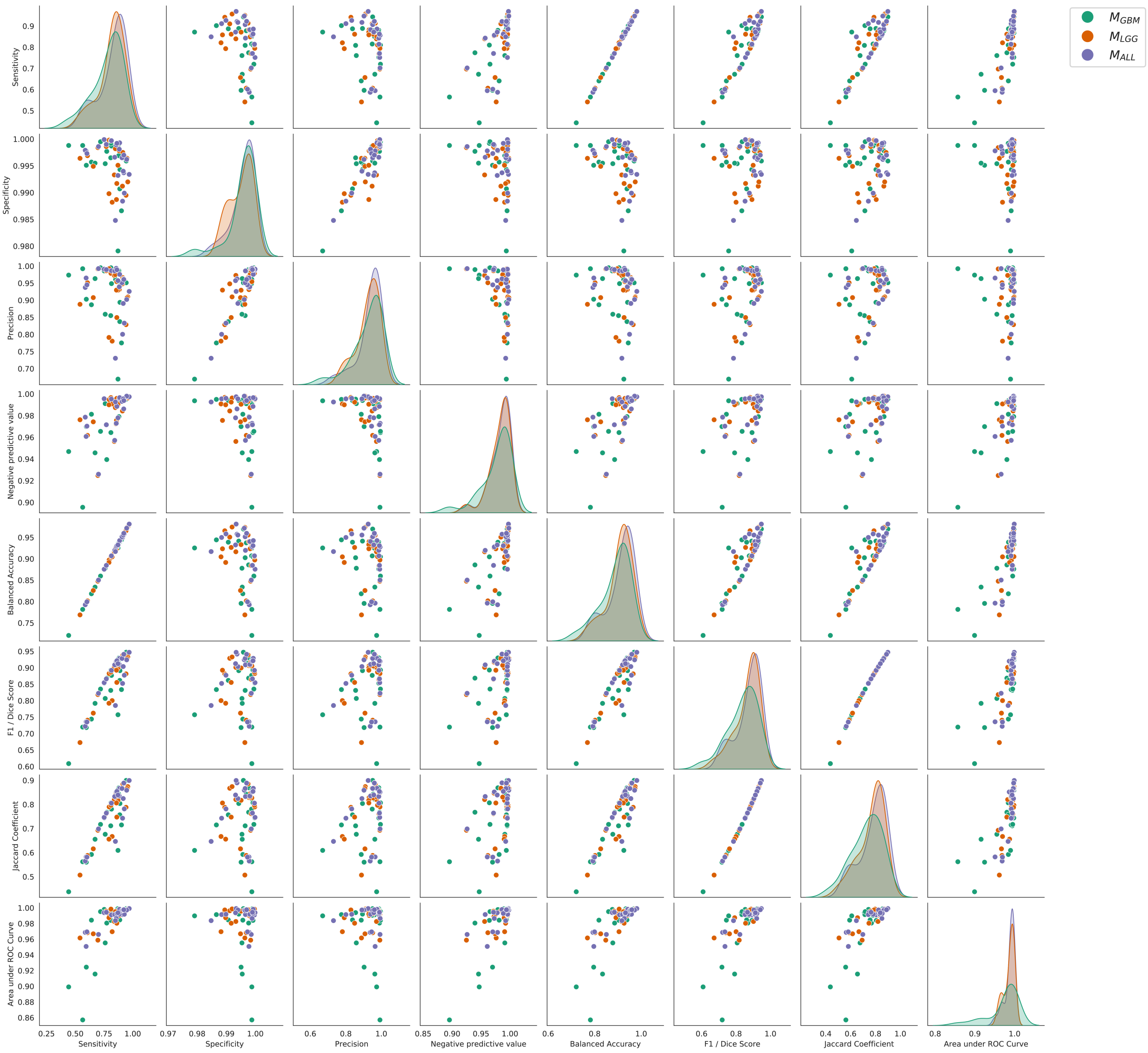

Figure S5. Pair plot of segmentation metrics of each patient in D_{LGG} across three ML algorithms. Marginal plots along the diagonal represent distributions for each metric across three ML algorithms. Dot plots on the off-diagonal represents the two-dimensional relationships between each pair of metrics. ML algorithms are annotated by colors.

4 SUPPLEMENTARY FIGURES: MODEL UNCERTAINTY

In the experiment on model confidence, we train Conformal Quantile Regressors that estimate the prediction interval upper and lower bounds. Here, we present example images of model predictions from D_{GBM} and D_{LGG} along with the model confidence.

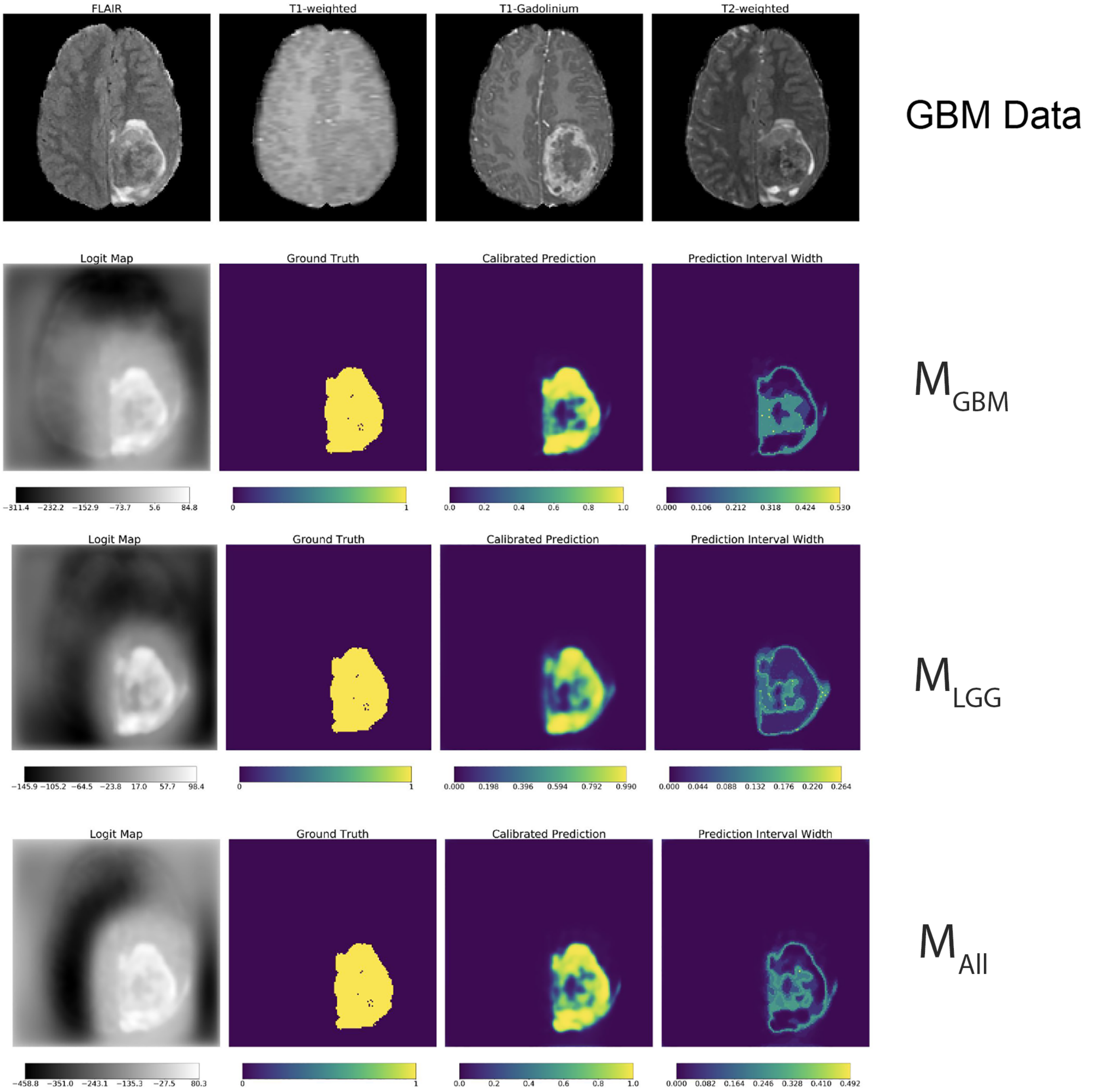

Figure S6. Model confidence plots for patient TCGA-08-0521.

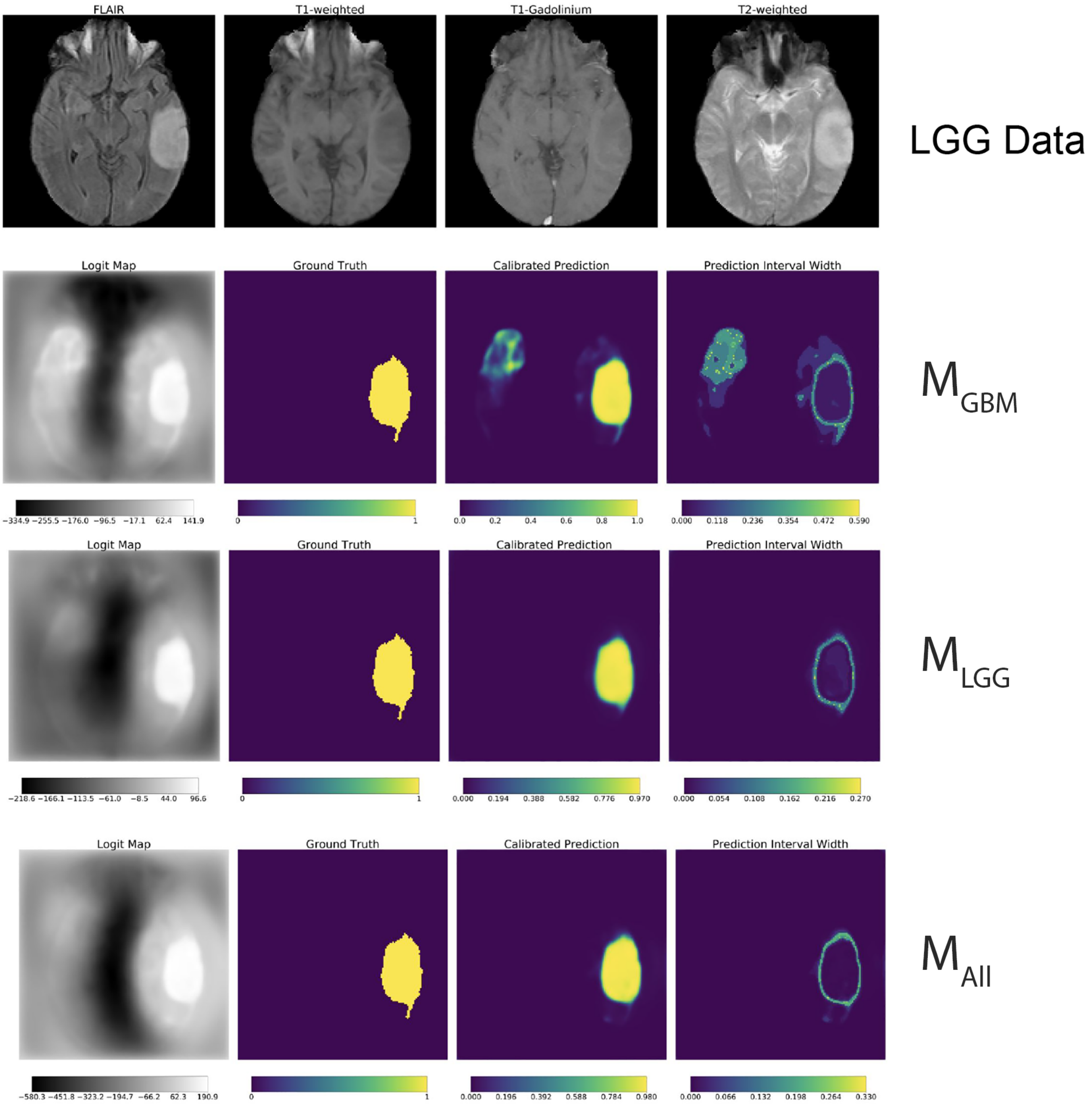

Figure S7. Model confidence plots for patient TCGA-CS-6290.
